# Questionable Research Practices, Low Statistical Power, and Other Obstacles to Replicability: Why Preclinical Neuroscience Research Would Benefit from Registered Reports

**DOI:** 10.1523/ENEURO.0017-22.2022

**Published:** 2022-08-02

**Authors:** Randall J. Ellis

**Affiliations:** Friedman Brain Institute, Department of Neuroscience, Addiction Institute of Mount Sinai, Icahn School of Medicine at Mount Sinai, New York, NY 10029

**Keywords:** metascience, questionable research practices, registered reports, replicability, reproducibility, statistical power

## Abstract

Replicability, the degree to which a previous scientific finding can be repeated in a distinct set of data, has been considered an integral component of institutionalized scientific practice since its inception several hundred years ago. In the past decade, large-scale replication studies have demonstrated that replicability is far from favorable, across multiple scientific fields. Here, I evaluate this literature and describe contributing factors including the prevalence of questionable research practices (QRPs), misunderstanding of *p*-values, and low statistical power. I subsequently discuss how these issues manifest specifically in preclinical neuroscience research. I conclude that these problems are multifaceted and difficult to solve, relying on the actions of early and late career researchers, funding sources, academic publishers, and others. I assert that any viable solution to the problem of substandard replicability must include changing academic incentives, with adoption of registered reports being the most immediately impactful and pragmatic strategy. For animal research in particular, comprehensive reporting guidelines that document potential sources of sensitivity for experimental outcomes is an essential addition.

## Significance Statement

For hundreds of years, scientists have written about different forms of poor scientific practice. In the past decade, scientists have shown that replicability of scientific findings is far from optimal across fields and likely impacted by questionable research practices (QRPs), along with related incentives to publish positive findings. Animal research is the arena for testing novel biomedical hypotheses and treatments before being studied in clinical trials. Understanding how different factors compromise replicability in preclinical neuroscience research, and solutions to mitigating them, is critical to identifying new neurologic and psychiatric treatments to improve the human condition, and reducing research waste. Two essential strategies to align scientific incentives with rigor are the adoption of registered reports and comprehensive reporting guidelines.

## Introduction

Replication of scientific findings is fundamental to the cumulative development and validation of theories, medical treatments, and methods. Over the past decade, large-scale investigations regarding replicability of published findings have been conducted in various scientific fields, including but not limited to psychology (Klein et al., 2014, 2018, 2019; Open Science Collaboration, 2015; Ebersole et al., 2016), cancer biology (Prinz et al., 2011; Begley and Ellis, 2012; Errington et al., 2021), social sciences (Camerer et al., 2018), economics (Camerer et al., 2016), and machine learning (Beam et al., 2020; Pineau et al., 2020; Table 1). These studies have demonstrated that replicability is alarmingly low in many fields of science. A 2016 survey by *Nature* showed that these issues are well known to scientists, 90% of 1576 researchers answered the question “Is there a reproducibility crisis?” with “Yes, a significant crisis” (52%) or “Yes, a slight crisis” (38%; Baker, 2016). It is estimated that at least $28 billion is wasted on irreproducible preclinical science annually in the United States alone (Freedman et al., 2015). Proposed factors in the perceived “replication crisis” include widespread questionable research practices (QRPs) such as publication bias (results affecting choices to publish or not, also known as the “file-drawer problem”), p-hacking (analytical flexibility with the goal of calculating a *p* < 0.05), and HARKing (“Hypothesizing After Results are Known,” also known as *post hoc* accommodation of theories; John et al., 2012), incentives imposed by a publish-or-perish academic culture that only emphasizes positive results to the exclusion of null findings (Nosek et al., 2012), low base rates of true effects (Wilson and Wixted, 2018), inadequacies of null hypothesis significance testing (NHST), and many others. Efforts to mitigate replicability issues include but are not limited to greater transparency of data and research methodology (e.g., experimental protocols, analysis code; Munafò et al., 2017), preregistration and registered reports (Chambers and Tzavella, 2022), specification curve analysis (also known as “multiverse analysis”; Patel et al., 2015), changing the standard threshold of statistical significance from 0.05 to 0.005 (Benjamin et al., 2018), adopting Bayesian statistics (Etz and Vandekerckhove, 2016), justifying α levels and sample sizes (Lakens et al., 2018; Lakens, 2021a), normalizing the publication of negative findings (Wasserstein et al., 2019), computer-generated results files (Lakens and DeBruine, 2021), and countless others.

**Table 1:** Statistics reported in six replication projects spanning cancer biology, economics, psychology, and social sciences

Reference	Field	Number of original positive outcomes	Effect size, originals, mean (SD) or median	Effect size, replications, mean (SD) or median	% replications statistically significant, same direction	% original point estimates in CI of replication
Klein et al. (2014)	Psychology	15^*a*^	0.745 (0.276)	0.887 (0.796)	80	26.67
Open Science Collaboration (2015)	Psychology	97	0.403 (0.188)	0.197 (0.257)	36	47
Camerer et al. (2016)	Economics	18	0.474 (0.239)	0.279 (0.234)	61	66.7
Klein et al. (2018)	Psychology	28	0.6	0.15	54	0
Camerer et al. (2018)	Social sciences	21	0.459 (0.229)	0.249 (0.283)	62	61.9
Errington et al. (2021)	Cancer biology	97^*b*^	6.15 (12.39)	1.37 (3.01)	43	18

Effect sizes for original and replication studies were reported as either means or medians. Effect sizes without a value in parentheses are medians. Confidence intervals are either 95% or 99%. Replications of original null outcomes are not considered here. SD = standard deviation; CI = confidence interval.

a Klein et al. (2014) replicated 16 original positive effects, but one did not have an original effect size reported and is not considered here.

b Errington et al. (2021) replicated 136 original positive effects, but only 97 were reported as numerical values; others were reported as images.

As a field, psychology has undertaken the deepest conversation about these issues and traveled farthest toward integrating potential solutions into scientific practice. Large-scale replication projects of psychological studies have been conducted that include multiple sites and sample sizes multiple times larger than the original studies (hence greater statistical power). In many of these replications, effects are much smaller than those in the original studies (Open Science Collaboration, 2015; Camerer et al., 2018; Klein et al., 2018). Replication projects in other fields show similar results. These replication projects are the most informative efforts to date about the extent of the replicability crisis and have galvanized conversations on how to improve various facets of scientific practice to mitigate it. Proposed solutions to these issues are applicable to many scientific fields beyond psychology, and are worth examining.

The goal of this commentary is to introduce neuroscience researchers to metascience by examining deficits of replicability demonstrated by the results of these large-scale replication projects, proposed sources of and solutions to mitigate them, and contextualizing their impact in particular on preclinical neuroscience research. I examine the impact of these issues on the quality of published work, specific solutions in scientific and institutional practice that can be made to combat them, and pragmatic implementations of these solutions that can benefit early career and senior researchers, funding sources, and academic publishers.

I begin by reviewing the literature on large-scale replication projects to quantify the gap between original studies and replications in terms of reported effect sizes, statistical significance, and other measures of replication success. I then examine a few potential contributors to this gap, and solutions to bridge it. This provides the foundation for examining replicability specifically within preclinical neuroscience, and what can be learned from efforts being made (primarily within Psychology) to mitigate these problems. Finally, I make the case for why any viable solution to improve replicability must include, and ideally begin with, preregistration in the form of registered reports. For animal research, comprehensive reporting of environmental and other conditions that can impact results is a critical addition. In each section, I will discuss how the specific issues being addressed impact preclinical neuroscience research.

A quick note about terminology: this commentary primarily deals with “replicability,” as opposed to “reproducibility” or “robustness,” the definitions of which are borrowed from a recent review (Nosek et al., 2021). Replicability refers to the ability to answer the same question with new data and obtain similar results compatible with prior studies. Reproducibility refers to the ability to answer the same question with the same data using the same analysis strategy and achieve the same result. Robustness refers to the ability to answer the same question with the same data using a different analysis strategy and achieve the same result.

## Large-Scale Replication Projects Demonstrate that Replicability Is Far from Optimal across Scientific Fields

In the past decade, replication studies in psychology, cancer biology, and other fields have demonstrated that replicability is often difficult to achieve (Table 1). Replicability is typically defined and described using the following criterion: (1) *p*-values that are below a particular α threshold (almost always 0.05); (2) effect size magnitude and directionality; (3) meta-analysis of original and replication effect sizes; (4) whether the point estimate effect size of an original study is within the confidence interval of the replication effect size (and vice-versa); (5) subjective assessments by replication teams. Replication studies can be designed in many different ways. The Open Science Collaboration published a replication of 100 studies originally published in 2008 in three prominent psychology journals (Open Science Collaboration, 2015), with less than half of replications being deemed successful.

The first two “Many Labs” replication studies, also in psychology, replicated classic and contemporary psychological effects published between 1936 and 2014 and showed similar results (Klein et al., 2014, 2018). Many Labs 4 replicated a single classic effect in 21 labs, and measured the effect of involvement of the original authors on replication success. Nine labs were advised by one of the authors of the original study, and 12 were not. Only 1/21 labs showed a statistically significant effect, and this lab was not advised by the original author.

Replication projects from other fields have not fared better. In 2011, scientists from Bayer published a replication of 67 preclinical cancer, cardiovascular, and women’s health studies (Prinz et al., 2011). Approximately 20–25% of original findings were completely in line with the replications, and 32% were replicated to some degree. In 2012, Amgen published a replication of 53 preclinical cancer studies considered “landmark” studies, and only six successfully replicated (Begley and Ellis, 2012). More recently, the Reproducibility Project: Cancer Biology repeated 50 experiments from 23 papers, testing 158 effects, where 136 of the original effects were positive (Errington et al., 2021). For original positive effects, 43% of replication effects were both statistically significant and in the same direction as the original, 49% showed null results, and 7% were significant in the opposite direction. For original null effects, 73% of replication effects are also null. Critically, 92% of all replications of positive effects had effect sizes smaller than the originals, mean replication effect size was 78% smaller than the mean original effect size, and median replication effect size was 85% smaller than the median original effect size.

Replications of findings from top journals are not much more successful. In 21 replications of social science studies published in *Nature* or *Science* (Camerer et al., 2018), 13 (62%) had a statistically significant effect in the same direction, and effect sizes were on average 50% of the original effect sizes.

These and other results demonstrate that replicability may not be ideal across multiple scientific fields, with replication effects being ∼50% smaller, and ∼50% showing statistical significance in the same direction as original studies.

What do these results mean for preclinical neuroscience? There are sparse replication projects in neuroscience generally, let alone preclinical neuroscience, but they are very much worth discussing. In one study, 41 visual field asymmetries (i.e., differential processing efficiencies between left and right visual fields) studied across nine experiments were replicated and yielded evidence of these processing asymmetries for emotions, faces, and words, but not stimuli with high or low spatial frequencies (Brederoo et al., 2019). With regards to robustness, a 2020 study showed that when 70 teams tested the same nine hypotheses using the same functional magnetic resonance imaging dataset, no two teams used the same workflow to analyze the data, leading to highly varied results (Botvinik-Nezer et al., 2020). While not a replication study per se, this study indicates that analytical flexibility could strongly impair replication success in neuroimaging studies. The only large-scale replication project focused on neuroscience, still in progress, is the #EEGManyLabs project, which aims to replicate findings from 20 of the most influential studies in the field in three or more independent laboratories, with experimental designs and protocols to be reviewed before data collection as registered reports (Pavlov et al., 2021).

Since there are no completed large-scale replication projects focused on neuroscience, one can only guess what the results would be. In the aforementioned replication projects, roughly half of effects reached statistical significance in the same direction as their original studies, and effect sizes were typically half those of the originals, though these results certainly do not necessitate that a neuroscience-focused project would show the same results, and replicability may vary widely across subfields of neuroscience.

A critical question asked by all is what are the sources of the lack of replicability seen across multiple fields. It is impractical to give an in-depth view of all factors, so I confine the discussion to QRPs, incentives within academia, misunderstanding of *p*-values, and low statistical power.

## QRPs and Academic Incentives

QRPs refer to actions taken in the design, analysis, or reporting of a scientific study that can confer a bias, usually in support of some desired conclusion on the part of the scientist (Banks et al., 2016). Here, I describe QRPs, evidence of their prevalence, and why scientists may engage in them.

- QRPs include but are not limited to selective reporting of hypotheses or results (e.g., ones that “work”), p-hacking (also known as “data dredging,” in an effort to present statistically significant *p*-values), HARKing, rounding of *p*-values, premature stopping or purposeful extension of data collection in hopes of reaching a desired conclusion, selective outlier removal, and data falsification. It is worth noting that HARKing is only problematic in the context of confirmatory research, not exploratory research where hypotheses are not being tested, but rather generated.
- QRPs have a long history. P-hacking has been described since at least 1830 in Charles Babbage’s *Reflections on the Decline of Science in England, and on Some of its Causes* (referred to as “cooking,” and Babbage also described selective outlier removal, what he referred to as “trimming”; Babbage, 1830).
- The prevalence of QRPs is difficult to know, but the evidence is not optimistic. A survey of 2155 psychologists for their estimates of the prevalence of and their own engagement in 10 QRPs showed self-admission rates ranging from 0.6% to 63.4%, with prevalence estimates 5–20% higher for all but one QRP (John et al., 2012).These results were replicated in a cohort of 277 Italian psychologists with a translated questionnaire (Agnoli et al., 2017), and largely agree with several other surveys of QRP engagement (for a summary, see Table S5 in a review by Nosek and colleagues; Nosek et al., 2021; see also Fanelli, 2009; Banks et al., 2016; Fiedler and Schwarz, 2016; Motyl et al., 2017; Krishna and Peter, 2018; Janke et al., 2019; Rabelo et al., 2020; Moran et al., 2021).
- Why do scientists engage in QRPs? The answer may be that academic research incentivizes them to (Edwards and Roy, 2017). Researchers advance their careers by securing grants and publishing papers in high-impact journals. High impact factor journals tend to publish positive data, with null findings typically considered failures or otherwise not useful. Because of the emphasis of academic journals on positive findings, scientists may engage in QRPs to obtain positive results and exclude null results (i.e., publication bias). To clarify, the current academic culture, where the two primary currencies are publishing high-impact papers and obtaining grants, does not alone incentivize engagement in QRPs, but rather the emphasis on positive findings from funding sources and journals in achieving these two ends.
- The effects of the emphasis on positive findings are many. One is how the frequency of published null findings has changed over time across fields. In a sample of 4656 papers across disciplines published between 1990 and 2007, full or partial support of hypotheses increased over 20% during this interval, with stronger trends in psychology/psychiatry, economics, and social sciences (Fanelli, 2012). In a sample of 2434 papers across 20 scientific fields, the percentage of papers reporting support for the tested hypothesis ranged from 70.2% to 91.5%, with psychology and psychiatry having the most (91.5%; Fanelli, 2010). For the sake of argument, suppose these trends do not indicate QRPs, but that science is improving in terms of proposing successful hypotheses and identifying true effects. The two problems with this supposition are the following: (1) this would contradict the observed deficits in replicability across fields; and (2) if this were true, it would mean science is being too conservative and not taking enough risks in making novel discoveries. Accounting for the incentives to publish positive results, the increasing percentage of reported positive results, and the issues of replicability observed across fields, I can suppose that a substantial percentage of published findings are false positives and that a large number of null findings are not published.
- Most journals do not encourage the submission of replications, and even if they do, editorial policies are often not followed (Martin and Clarke, 2017; Nieuwland, 2018).
- The percentage of biomedical and life-science research articles retracted because of fraud increased 10-fold between 1975 and 2012, and misconduct accounted for 67.4% of retractions as of 2012, including fraud (43.4%), duplicate publications (14.2%), and plagiarism (9.8%; Fang et al., 2012).
- What does the public think? As the primary funder of science, it is worth examining how the public views scientists who engage in QRPs and what repercussions, if any, are considered reasonable. Pickett and Roche conducted surveys of American adults (*n* = 821) using Amazon Mechanical Turk about what penalties should be imposed on scientists who engage in two specific QRPs: data falsification/fabrication, and selective reporting (Pickett and Roche, 2018). For data falsification, over 90% of respondents said scientists should be fired and banned from receiving government funding, and 66% said it should be considered a crime. These results are not so surprising as this is quite an egregious practice, and the surveys mentioned previously support this behavior being rare. More relevant are the perceptions of selective reporting: 63% of respondents advocated firing, and 73% for a ban on receiving funding. Only 37% said selective reporting should be considered a crime. If the punishments specified by the public were carried out, there may be a large purge of the scientific workforce. Recent work has begun to sketch the contours of a criminological framework for studying research misconduct (Faria, 2018; Bülow and Helgesson, 2019).

In sum, QRPs are widespread and take many forms including p-hacking, selective reporting, and HARKing. QRPs reduce the quality of the scientific literature and are incentivized in the current academic climate where journals prioritize positive findings, and publications in these journals are essential to winning grants and advancing one’s career overall. The definition of what is publishable in prestigious journals is having a detrimental effect on research quality, and as the academic job market is competitive (Langin, 2019), this may lead to a race-to-the-bottom in terms of engagement in QRPs. Surveys of the public advocate for professional and legal punishment for engaging in not only severe QRPs like data falsification, but also selective reporting, which surveys show is quite common.

I contend that QRPs and the incentives for their engagement (i.e., publishing decisions based on positive/significant findings) represent the single largest obstacle to improving replicability in science and living up to the description of science as a self-correcting enterprise. While there are no published surveys of neuroscientists related to engagement in QRPs, the same incentive structures described are arguably inherent to neuroscience, and the prevalence of QRPs in neuroscience remains an open question.

## Misunderstanding of *P*-Values

Aside from QRPs increasing false positive rates, another major obstacle to improving replicability in science is the incorrect use and understanding of *p*-values (Greenland et al., 2016). A *p*-value represents the probability of obtaining a result, or one more extreme, on the condition of a point hypothesis being true (Benjamin et al., 2018). Goodman names 12 common misconceptions of the *p*-value (Goodman, 2008), four of which are: (1) if *p* = 0.05, the null hypothesis has only a 5% chance of being true; (2) if *p* ≥ 0.05, there is no difference between experimental groups; (3) *p* = 0.05 means that I have observed data that would occur only 5% of the time under the null hypothesis; (4) with a *p* = 0.05 threshold for significance, the chance of a Type I error will be 5%. All 12 are worth examining, but I will explain these four here. (1) is false because the *p*-value has nothing to say about the probability of the null hypothesis being true. It is already assumed that the null hypothesis is true, and the *p*-value refers to the probability of obtaining data, or data more extreme, under this assumption. (2) is false because *p* ≥ 0.05 only means that a null effect is statistically consistent with the observed results, but so are the effects within the confidence interval. It does not make the null effect the true or most likely effect. (3) is false because *p* = 0.05 means that I have observed data that would occur, along with data more extreme, only 5% of the time under the null hypothesis. Finally, (4) is not necessarily either true or false. If you have a null hypothesis that you know to be true, then the probability of a Type I error is actually 5% if the assumptions of the statistical test used are met (Wilcox, 2016). If you know the null is false, there is no Type I error because any positive result would be a true positive. This point also does not address scenarios where multiple comparisons are made and the Type I error rate for any individual comparison is increased beyond 5%, requiring statistical correction.

Trafimow and Earp give a short checklist of things the *p*-value does not tell us (Trafimow and Earp, 2017): (1) the probability of the null hypothesis being true; (2) the probability of the alternative hypothesis being true; (3) a valid index of effect size; (4) a valid index of the degree of generalizability of the findings; (5) a valid indicator of the probability of replication.

Theoretical and practical recommendations to improve the integrity and utility of the use of *p*-values relate to more rigorously linking theoretical claims with experimental models to justify dichotomous claims (Tunç et al., 2021), confirming that researchers’ questions are answerable using *p*-values (Lakens, 2021b), and justifying α levels (Lakens et al., 2018) and sample sizes (Lakens, 2021a). Others have proposed changing the customary α threshold from 0.05 to 0.005 (Benjamin et al., 2018), abandoning the concept of statistical significance altogether and treating *p*-values with a less dichotomous and more continuous view (Amrhein et al., 2019; McShane et al., 2019), and supplementing or replacing frequentist statistics with Bayesian statistics (with the Bayes factor as the replacement for the *p*-value; Greenland and Poole, 2013; Wagenmakers et al., 2018).

I do not take a hard stance on any of these proposals because in my view, the incentives of research represent the largest barrier to improving replicability. Regardless of which *p*-value threshold is used or whether the *p*-value is replaced by the Bayes factor, incentives to publish positive results will lead to any statistical decision rule that impacts publishing decisions and career advancement being gamed and manipulated. Researchers should be better-educated about what exact information different statistical approaches can provide, the dangers of data dredging without multiple testing correction or transparent reporting of all comparisons, how to justify study parameters (sample size, power), and other aspects of proper use and potential pitfalls of statistical practice. But with the current incentives for positive findings, no set of purely statistical norms will prevent researchers from identifying opportunities for analytical flexibility (e.g., data dredging, outlier removal) that can both yield positive results and be minimally justifiable for publication without being outright fraud.

How does the misunderstanding of *p*-values manifest in preclinical neuroscience? One major way is in how interaction effects are studied (Nieuwenhuis et al., 2011). As an example, suppose you were studying the effect of reward-associated cues on neural firing in the ventral tegmental area (VTA) in wild-type and mutant strains of mice. You discover that the difference in VTA firing between the cued and un-cued conditions is statistically significant in wild-type mice (*p* < 0.05), but not the mutants (*p* > 0.05). Declaring this a strain-specific effect is incorrect, albeit common, because the two strains have not been directly compared in, for example, a two-way analysis of variance. “When making a comparison between two effects, researchers should report the statistical significance of their difference rather than the difference between their significance levels” (Nieuwenhuis et al., 2011).

Another common example of this error is when the expression of a gene is compared between two experimental groups within multiple brain regions, where the group-level comparison is significant in one region but not the others, and this is labeled a “region-specific effect.” This is incorrect for the same reason, the significance levels of each region are being compared, but not the differences of expression.

A specific case of this error involves the reporting of sex differences. If an experimental treatment shows a statistically significant effect in, for example, females but not males, this is often presented as a sex-specific effect. This is again incorrect, and bibliometric analyses show that this error is committed in the majority of articles claiming sex differences (Garcia-Sifuentes and Maney, 2021). As the measurement of sex differences has been a component of NIH guidelines since 2014 (Clayton and Collins, 2014), the prevalence of these statistical errors demands the neuroscience community’s attention in quelling them.

Other cases in neuroscience relate to how data from specific experimental techniques are analyzed. Temporal correlations in neuronal data from electrophysiology can lead to spurious identification of action-value representations, and analytical and experimental techniques used to parse these action-value representations from other decision variables are often inadequate (Elber-Dorozko and Loewenstein, 2018). Specific to human studies, Héroux and colleagues examined 1560 papers by 154 survey respondents who conduct electrical brain stimulation research, and found that 25% admitted to adjusting statistical analyses to obtain positive results (43% claimed to be aware of others doing this; Héroux et al., 2017). Luck and Gaspelin demonstrate how analytical flexibility in event-related potentials studies can lead to statistically significant yet incorrect results and describe steps that can be taken to prevent this (Luck and Gaspelin, 2017).

In sum, *p*-values are often misinterpreted, and these misinterpretations affect the accuracy of results reporting in neuroscience and many other fields.

## Low Statistical Power

Aside from misinterpretations and misuses of *p*-values, another basic statistical consideration that is often inadequate is statistical power.

Statistical power is the probability of not committing a Type II error (false negative), and the complement of the Type II error rate β (statistical power is 1-β). Restated, statistical power is the probability that a point hypothesis will be rejected (by obtaining a statistically significant result) when it is false, and calculating it depends on the α threshold, sample size, and a smallest effect size of interest (Cohen, 1992; Morey and Lakens, 2016). The typically prescribed level of statistical power is 80% for a given study design, smallest effect size of interest, and α threshold. Low statistical power to detect a smallest effect size of interest introduces two important vulnerabilities to a scientific study: (1) higher probability of Type II error, and (2) lower likelihood that a statistically significant result reflects a true effect (Button et al., 2013a). (2) is less discussed than (1), and is illustrated in the following formula linking statistical power with positive predictive value (probability that a statistically significant effect reflects a true effect):

PPV = ([1−β] × R)/([1−β] × R +α),

where (1-β) is statistical power, β is the Type II error rate, α is the Type I error rate, and R is the prestudy odds of an effect not being a null effect among the effects measured (Button et al., 2013a).

There is evidence that a substantial portion of published studies have low statistical power to detect their associated effect sizes. Dumas-Mallet and colleagues examined 660 meta-analyses published between 2008 and 2012 of biological, environmental, and cognitive/behavioral measures related to neurologic, psychiatric, and somatic disease in humans (Dumas-Mallet et al., 2017). Statistical power of their composing studies was measured assuming the meta-analytic effect sizes were the true population effect sizes. ∼50% of studies had statistical power between 0% and 20%, and 18% of studies had 80% power or greater. When only analyzing studies from meta-analyses with statistically significant effects (*n* = 420), ∼50% of studies had statistical power between 0% and 40% and <30% of studies had 80% power or greater.

Discussions of low statistical power in published literature extend at least since 1962, when Cohen examined the power of studies published in the 1960 issue of the *Journal of Abnormal and Social Psychology*, and found that average power to detect small, medium, and large effects was 18%, 48%, and 83%, respectively (Cohen, 1962). In 1989, Sedlmeier and Gigerenzer examined articles published in the same journal in 1984 and found that mean power to detect a medium effect decreased to 37%, in addition to reviews of other journals showing similar statistics as Cohen’s original study (Sedlmeier and Gigerenzer, 1989). In a small 2014 survey asking how scientists justified sample sizes, 58.5% answered “The number is typical for my area” or “I used the same sample size as another study” (Vankov et al., 2014). Only 9.6% of respondents answered “I ran a formal power analysis.”

In neuroscience, studies tend to be underpowered to detect the effect sizes that are published at an α threshold of 0.05 (Button et al., 2013a; Nord et al., 2017). Button and colleagues analyzed 49 meta-analyses published in 2011 (composed of 730 original studies) and found that the median statistical power was 21%, under the assumption that the meta-analytic effect size was the true population effect size (Button et al., 2013a). Nord et al., re-analyzed these data using Gaussian mixture models to quantify heterogeneity of statistical power across neuroscience subfields and found that median statistical power ranged from 11% to 57% (genetic: 11%; treatment: 20%; neuroimaging: 32%; neurochemistry: 47%; psychology: 50%; miscellaneous: 57%), highlighting candidate gene studies as having particularly low power (Nord et al., 2017). This large set of studies also did not show a statistically significant correlation between statistical power and impact factor (Brembs et al., 2013).

It is critical to also mention the evidence that meta-analytic effect sizes are biased upwards because of publication bias and selective reporting (Kvarven et al., 2020). Kvarven and colleagues compared 15 meta-analyses to 15 multilab replications of the same effects and found that 12/15 (80%) meta-analyses had statistically significantly larger effect sizes than their corresponding multilab replications (3/15 did not differ). If this trend is true for the meta-analyses examined by Dumas-Mallet and colleagues and Button and colleagues then their *post hoc* estimates of statistical power are likely biased upwards.

Some scientists make the error of neglecting statistical power if they observe large effects, because they assume that if a large effect is observed while underpowered to detect it, then a higher-powered study would yield an even larger effect. Loken and Gelman describe this fallacy as “assuming that that which does not kill statistical significance makes it stronger (Loken and Gelman, 2017).” This is wrong for two reasons: (1) if there was analytical flexibility present, this would raise the probability of statistically significant effects that do not reflect true effects (i.e., inflated false positive rate); and (2) statistical significance in a low-power, noisy setting is not strong evidence for the sign or magnitude of a true effect because these data typically have high standard errors. These notions are supported by simulations of the impact of measurement error on effect size when sample sizes range from 50 to 3000, demonstrating that noise can exaggerate effect size estimates in low N studies (Loken and Gelman, 2017). Low N studies have much less precise estimates of effect size and can result in what Gelman and Carlin called type M (exaggerated magnitudes of effects) and type S (when the sign of an effect is incorrect) errors (Gelman and Carlin, 2014). Because low sample sizes lead to greater standard errors around the mean, if a measured effect is statistically significant, it necessarily must be exaggerated (Vasishth et al., 2018). Larger sample sizes reduce standard errors and in turn yield more precise estimates of effect size. It is worth mentioning that this reduction of standard errors resulting from increased sample sizes raises the probability of statistically significant results for small effects, and this point was raised in response to the study of statistical power in neuroscience conducted by (Button et al., 2013b; Quinlan 2013). However, this point overlooks that the effect sizes of statistically significant effects in low-powered settings are likely exaggerated, and it is arguably most important for a field to precisely estimate effect size magnitudes, rather than only if they are statistically significant or not (Button et al., 2013b). Knowing precise effect sizes will enable a field to know how interesting an experimental intervention is, and whether it is worth following up on.

Along with the studies of statistical power across neuroscience from Button, Nord, and colleagues, Szucs and Ioannidis examined effect sizes and statistical power in 26,841 statistical tests from 3801 cognitive neuroscience and psychology papers published between 2011 and 2014 in 18 specialty journals (Szucs and Ioannidis, 2017). Across these studies, the median effect size was 0.93 (interquartile range: 0.64–1.46), and median power was, for differently sized effects: 0.12 (small, Cohen’s *d *=* *0.2), 0.44 (medium, *d *=* *0.5), 0.73 (large, *d *=* *0.8). Importantly, cognitive neuroscience studies tended to have larger effect sizes and lower degrees of freedom (indicating low sample sizes), and these were associated with publication in higher impact journals. Indeed, this tendency yielded negative correlations between journal impact factor and median statistical power for small (*r* = −0.42 [−0.63; −0.09]), medium (−0.46 [−0.71; −0.09]), and large (−0.45 [−0.77; −0.02]) effects.

The Editorial board of the *Journal of Neuroscience* recently recommended that studies be designed with two experimental stages: an initial exploratory stage that may provide evidence for an effect, albeit a likely inflated one because of the twin issues of low sample sizes and low statistical power, and a second stage focused on most precisely estimating the magnitude of this effect (Society for Neuroscience, 2020). Here, the first stage can be powered to only detect medium and large effects, but the second stage should be powered to detect small effects. Not only would this design entail a replication of an initially measured effect, but it also coincides with Fisher’s original recommendation for how to use the *p*-value threshold of 0.05 (that an experiment should be repeated) (Goodman, 2008). Further, it would refine dichotomous experimental questions of whether an effect exists or not, to precisely estimating the magnitude and direction of said effects.

## Issues Specific to Animal Research

Replicability is a serious and widespread issue across scientific fields, and there is a significant contribution of social (QRPs, inappropriate incentives) and statistical (misunderstanding of *p*-values, low statistical power) factors to low replicability. But how do these issues impact animal research in particular, as a large portion of neuroscience is conducted in animals, and certainly that which aims to establish theoretical foundations or clinically translational constructs?

There is evidence that the animal research literature tends to be under-powered. In 2003, Jennions and Møller conducted a survey of behavioral ecology and animal behavior studies (Jennions and Møller, 2003). Across 697 papers in 10 journals, the power to detect small, medium, and large effects was 13–16%, 40–47%, and 65–72%, respectively, with 10–20% of studies exceeding 80% statistical power. A 2018 survey of 410 experiments across 122 articles in the rodent fear conditioning literature yielded a mean effect size of 37.2% for well-powered, statistically significant effects, and only 12.2% of studies had 80% statistical power or greater (Carneiro et al., 2018).

To combat low statistical power, the field must increase sample sizes, which contrasts with the 3Rs of animal research (Replace, Reduce, Refine; Würbel, 2017). How is the statistical power and replicability of animal studies supposed to improve if animal researchers are encouraged to use as few animals as possible? While minimizing animal suffering is essential, low statistical power reduces the scientific value of animal studies. However, I argue that increasing the statistical power of animal studies is not currently the highest priority for improving the quality of preclinical animal research. I argue that the most important issue with preclinical animal research is the incentive structure that leads to engagement in QRPs. Animal research is essential to alleviating human suffering and has produced myriad breakthroughs and interventions that have immensely improved the human condition, but to inflict discomfort on animals *and* engage in QRPs is strongly unethical.

The publication rate alone in animal research is alarming. Van der Naald and colleagues in their “plea for preregistration,” examined 67 animal study protocols approved by the animal ethics committee at the University Medical Center Utrecht between 2008 and 2009 that were conducted to completion (van der Naald et al., 2020). First, only 46% (31/67) of these protocols had a resulting publication, and this increased to 60% (40/67) when conference abstracts were included, already an indication of publication bias. Out of 5590 animals used in all 67 protocols, only 26.3% (1471) were published that were associated with these 40 protocols. Limiting the scope to these 40 protocols, only 33% of animals were published (1471/4402). The publication rate specifically for small animals (mice, rats) was 23% (1190/5014) and the rate for large animals (pigs, dogs, sheep) was 52% (299/576). 115.3 million laboratory animals are estimated to have been used worldwide in 2005 alone (Taylor et al., 2008; although the authors say this “is still likely to be an underestimate”), and assuming van der Naald’s percentage (26.3%) is representative, this means 85 million animals from 2005 are unpublished. The number of laboratory animals used is surely higher presently.

Other studies have shown higher but nevertheless alarmingly low rates of publication for animal research. Wieschowski et al., found a publication rate of 67% across 158 animal research protocols at two German university medical centers, and this decreased to 58% when doctoral theses were excluded (Wieschowski et al., 2019). A Dutch survey conducted in 2011 of 421 animal researchers working at not-for-profit institutes showed estimates that 50% of animal research is published (interquartile range: 35–70%; Riet et al., 2012).

It is worth nothing that low publication *rates* do not necessitate publication *bias* or selective reporting–perhaps some of these animals were used for pilot studies, testing new techniques, studies were not continued after receiving revisions, or other reasonable activities such as wanting to publish null data but being denied by journals. But even if I consider a liberal adjustment that accounts for double the percentage of animals used according to van der Naald and colleagues (52.6%), this still means 54.7 million animals are unpublished and unaccounted for from 2005 alone. The exact magnitude of the contribution of publication bias to animal publication rates is difficult to know because it requires knowing the exact reason for publication or nonpublication of specific animals. However, there is evidence from meta-analyses of animal research and surveys of animal researchers about the presence of publication bias. Across 21 meta-analyses assessing publication bias in animal research, evidence was found in 62% (13/21), though the methods for statistically assessing publication bias are imperfect (e.g., Egger’s test for funnel plot asymmetry, “trim-and-fill” analysis; Korevaar et al., 2011). Across 16 systematic reviews (composed of 525 studies) of interventions for acute ischemic stroke tested in animals, 2% of publications reported no effects on infarct volume, 1.2% reported no statistically significant findings, and Egger’s regression and trim-and-fill analysis identified publication bias in 16 and 10 reviews, respectively, with publication bias potentially accounting for 1/3 of reported efficacy (Sena et al., 2010). Further, a survey of 454 animal researchers estimated that 50% [median; 32% (Q1)−70% (Q3)] of animal experiments are published (Riet et al., 2012), and that the “important causes of non-publication” are (in descending order; 5-point scale): “Lack of statistically significant differences (‘negative’ findings)” (4; 4–5), “Instrumentation/technical problems” (4; 3–4), “Many studies are seen as pilot studies only” (3; 3–4), “Loss of interest” (2.5; 2–3), and “Lack of time to write manuscripts” (2; 2–3). These findings agree with the anonymous survey of psychologists mentioned earlier that showed a 45.8–50% self-admission rate and 60% prevalence estimate of “Selectively reporting studies that ‘worked’” (John et al., 2012). Overall, animal publication rates are low, and whether findings are positive appears to strongly impact publication decisions, giving support to the notion that publication bias is widespread in animal research.

These grim statistics illustrate the low publication rates, with some contribution of publication bias, in animal research. Worse still, one can only guess the prevalence of other QRPs in the animal research studies that actually make it to publication. For these reasons, I contend that before the scientific community can focus on increasing sample sizes to mitigate low statistical power, it is incumbent on us to change the incentives of the field to publish more of the animals the field is currently using and cease engagement in QRPs more generally. It is bad enough for 61/100 psychology studies with human participants who are not harmed in any way to not replicate Open Science Collaboration (2015), but it is much worse for studies conducted with animals where potentially 75% of them are not even published, with some percentage of the remaining 25% tainted by the presence of other QRPs. If animal studies had to be preregistered in the same manner as clinical trials (van der Naald et al., 2022), with primary outcomes and analysis plans, it is possible this would lead to a decline in the rate of published positive results, as it has in clinical trials (Kaplan et al., 2015).

A case can be made for increasing sample sizes to reach adequate levels of statistical power, but increased sample sizes in the presence of QRPs is even more unethical than underpowered QRP-ridden studies. Animal research will be more scientifically valuable if incentives are shifted away from positive results, and toward honestly and transparently using animal models to answer important questions with rigorous methodology. With the amount of trust given to researchers that conduct animal research, engaging in QRPs is unethical and unfortunate, but is a sad result of the incentives for positive findings rather than well-conducted experiments. Individual researchers and laboratories are not to blame for these problems as they reflect the norms and traditions that have coalesced over time, with the scientific enterprise as a whole to be held accountable for their creation and hopefully, rectification.

Other obstacles to high-quality animal research include low prevalence of blinded outcome assessment, randomization, statements of conflicts of interest, and sample size calculations. In 2671 studies published between 1992 and 2011 of drug efficacy in animal models of various diseases identified in systematic reviews, blinded assessment of outcome was reported in 788 publications (29.5%), randomization in 662 (24.8%), a statement of conflict of interest in 308 (11.5%), and a sample size calculation in 20 (0.7%; Macleod et al., 2015). Encouragingly, blind outcome assessment increased from 16.3% in 1992 to 39% in 2011, randomization from 14% to 42%, and conflict of interest reporting from 2.3% to 35.1%. Sample size calculations did not change across time, and this is a clear contributor to low statistical power, and in turn, low replicability. Examining the relationship between the presence of these practices and journal impact factor, reporting conflicts of interest was associated with a 2.6 higher impact factor, but randomization was associated with a 0.4 lower impact factor. There were no significant relationships for blinded outcome assessment or sample size calculation.

These issues with animal research are general, not specific to neuroscience, but should give neuroscientists pause in considering how they may impact their own studies.

## Sensitivity of Results in Animal Research: Generalizability and Replicability

If a replication experiment does not successfully recapitulate the findings of an original study, this may be partially explained by the original study’s findings not generalizing to specific conditions that were varied in the replication experiment. For example, there is a substantial literature documenting the sensitivity of results in animal research to various factors such as lab site, shipping versus in-house rearing, cage enrichment, housing temperature, protein and fat content of chow, animal strain, complex gene-environment interactions, and many others (Hylander and Repasky, 2016; Jarvis and Williams, 2016; Kafkafi et al., 2018). These issues may lead to results that do not replicate across labs or are statistically significant in opposite directions, and pose significant barriers to replicability more generally. While the issues I have already discussed (e.g., QRPs, misunderstanding of statistics, low power) undoubtedly contribute to them, these problems naturally accompany animal research and represent interesting scientific questions that will serve the scientific community by answering and understanding them. For example, if a particular effect is true and reproducible in one strain of rat but does not hold in another, this is not an issue of replicability, but of generalizability (Redish et al., 2018; Yarkoni, 2022). The general distinction is that replicability refers to how robust an effect is in terms of direction, magnitude, and statistical significance when the same experiment is re-run, and generalizability refers to how robust an effect is when certain parameters of an experiment (e.g., animal strain, housing temperature) or analysis (e.g., covariates, software used) are varied. Questions of generalizability relate to the sensitivity of conditions that can produce different results and enable a deeper understanding of the physiological and environmental variables of the models used and the experiments conducted.

As another example, it is known that housing temperature can affect antitumor responses (Hylander and Repasky, 2016), and if it can repeatedly be shown that housing temperature affects other results, this is again not an issue of replicability, but rather a scientific question about how housing temperature can affect the results of these experiments. These sensitivities represent scientific questions about the generalizability of findings, whereas QRPs and low statistical power directly harm replicability and the quality of scientific studies, only enhancing the difficulties raised by problems of generalizability that are often biological in nature, depending on the problem. It is critical to mention that issues of replicability (e.g., QRPs, low power) only compound with the problems of generalizability. The advantage of assessing generalizability in preclinical research is that the variables which researchers may incorrectly take for granted to generalize (e.g., temperature, animal strain, lighting sensitivity) are controllable and testable for their effects. The high controllability of animal experiments is both a blessing and a curse, for the ability to control extraneous factors and identify biological mechanisms beyond what can ethically be done in humans, with the caveat that many factors (co-housing, food/water, strain) may still compromise the replicability and generalizability of results.

## Case Studies in Neuroscience of Replicability/Generalizability Issues

I will now describe a few case studies in neuroscience that document how environmental and other experimental factors can influence results and indicate barriers to generalizability. A well-known case is that of Crabbe, Wahlsten, and Dudek (Crabbe et al., 1999) where six behaviors were tested in mice in three different labs, with efforts to standardize apparatus, testing protocols, and animal husbandry, using the same inbred strains, along with one null mutant strain. These behaviors were: locomotor activity in an open field (day 1; D1), elevated plus maze (D2), rotarod (D3), swimming to a visible platform (D4), locomotor increase after cocaine injection (D5), and preference for ethanol versus tap water (D6). To assess the effects of animal shipping, all three labs compared shipped mice to mice reared in-house. Comparing results across the three sites, there were strong and statistically significant effects for distance traveled and number of vertical movements in the open field, arm entries and time spent in the open arms of the plus maze, and body weight [partial ω^2^ (effect size) in a complete factorial ANOVA ranged from 0.157 to 0.327, all *p* < 0.00001]. Additionally, there was a strong and significant interaction of genotype and site for arm entries in the plus maze (partial ω^2^ 0.21, *p* < 0.00001), and weaker interaction effects on cocaine locomotor increase and body weight (partial ω^2^ 0.086 and 0.071 respectively, *p* < 0.001). Encouragingly, there were no statistically significant differences across sites for water maze escape latency or alcohol consumed. There was an effect of shipping versus local rearing on water maze escape latency, though this effect was weak and would likely not pass statistical correction. Interaction of genotype and shipping showed no statistically significant effects. Responses to this article highlighted the impact of social dominance hierarchies among group-housed mice on behavior, substrains and modifier genes in knock-out mouse colonies, differences in local tap water and Purina diet composition, and other potential sources of variation.

Wahlsten et al. (2006) compared behavioral data from inbred mouse strains across five decades and multiple laboratories and showed high stability for ethanol preference and locomotor activity, but large variance for elevated plus maze exploration, even across sites within the same university.

Richter and colleagues examined how systematic heterogenization of behavioral experiments affected replicability (Richter et al., 2011). Six laboratories each ordered 64 female mice of two inbred strains (C57BL/6NCrl, DBA/2NCrl), and conducted five behavioral tasks (barrier test, vertical pole test, elevated zero maze, open field, novel object test), under two experimental contexts (standardized or heterogenized). In the standardized condition, mice were tested at 12 weeks old, with only nesting material in their cages. In the heterogenized condition, mice were tested at either 8 or 16 weeks old, with either shelter or climbing structures in their cages. In both standardized and heterogenized conditions, order of tests, protocols, animal supplier, number of animals per cage, housing period before testing, cage position within racks, and cage change interval were all kept constant. Left to vary were local tap water, food, nesting material, cage size, testing room (layout, humidity, lighting, temperature), apparatus and tracking software, identification method (e.g., ear punches, fur markings), test time of day, and experimenter. In the standardized condition, 22/29 variables measured across the five behavioral tasks showed statistically significant effects of laboratory (*p*s ≤ 0.001). In the heterogenized condition, 23/29 variables showed these effects. Additionally, effects of strain were observed in the vast majority of measurements (not surprising given these two strains are known to differ behaviorally), along with interactions of lab and strain. In some cases, directionality of differences between C57BL/6NCrl and DBA/2NCrl differed by lab. It is worth noting that while multiple-testing correction is not shown in this study, the vast majority of significant effects had *p*-values below 0.001, indicating that many would pass correction, but the true number is unknown.

A highly instructive and valuable recent case comes from the International Brain Laboratory, where 140 mice in seven labs from three countries were trained on a modified two-alternative forced-choice task (International Brain Laboratory et al., 2021). While the learning rates for the task differed across labs, after reaching stability the mice showed marked similarities in decision-making and reliance on visual stimuli, past correct and incorrect trials, and estimates of prior probabilities for stimuli. This study demonstrates that large samples collected across multiple laboratories, with open preregistration and standardization of experimental protocols and analyses can yield replicable results. It is worth noting that along with behavioral apparatus, training protocols, hardware, software, and analysis code, also standardized were the protein and fat content ranges of the chow, mouse strain and providers, handling protocols, cage enrichment (nesting material and house, at minimum), weekend water (measured or with citric acid), and other variables. While it is possible that varying these factors would have impacted the results, particularly mouse strain (Crabbe et al., 1999), the space of variation explored gives credence to the generalizability of these results.

These and other studies demonstrate that variability in results can occur across laboratories conducting the same experiment. Discussions of systematic heterogenization are of continued interest and provide insight into how experimental designs can be modified to increase the generalization of individual studies without requiring larger sample sizes (Voelkl et al., 2020). One issue highlighted is that abundant standardization of experimental, environmental, and other factors, while potentially improving replicability across experimenters and sites, restricts the “inference space” that a study’s findings can be generalized to, and this is an important issue of discussion for animal researchers.

Less surprisingly, variability across strains is also prevalent across myriad phenotypes. Jung and colleagues discovered that Fisher 344 and Wistar-Kyoto rats differ in their reactivity to recent and reinstated fear memories, and showed overlapping and divergent blood transcriptome profiles, with strain-specific differentially expressed genes exhibiting different functional enrichments and co-expression (Jung et al., 2020).

Jin et al. (2017) discovered that citalopram reduces immobility time in the forced swim and tail suspension tests in DBA/2J mice but not C57BL/6J mice, with fluoxetine showing opposite results. Serotonin transporter affinity for citalopram was 700 times higher in DBA/2J mice, and fluoxetine 100 times higher in C57BL/6J mice. These same drug-strain effects were observed when measuring the effects of high-dose citalopram or fluoxetine on [^3^H]5-HT uptake in mouse cortical synaptosomes. Paroxetine showed consistent effects across strains.

Cabib and colleagues discovered that C57/BL6J and DBA/2J mice show opposite effects in place conditioning (preference and aversion, respectively) for amphetamine, but that food restriction reverses the aversion seen in the DBA/2J mice (Cabib et al., 2000). These and other results (Nguyen, 2000; O’Connor et al., 2021) yield valuable insights about the generalizability of effects observed using preclinical models that model aspects of psychiatric disorders.

Kafkafi and colleagues have written an excellent discussion of replicability, sensitivity, and interpretation of animal research (Kafkafi et al., 2018), along with several others (Landis et al., 2012; Crabbe, 2016; Jarvis and Williams, 2016; Percie du Sert et al., 2020; Voelkl et al., 2020; Drude et al., 2021; Hunniford et al., 2021; Lewis et al., 2021).

Not only can these genetic and environmental factors impact the replicability of animal research, they can also impede the translatability of animal findings to humans (Ioannidis, 2012; Lapchak et al., 2013; Garner, 2014; Kimmelman et al., 2014; McGonigle and Ruggeri, 2014). In a comparison of preclinical, early phase, and Phase 3 clinical trials of neuroprotective agents for acute stroke, 69.08% of preclinical studies were positive, in contrast to 32% of early clinical and 6% of Phase 3 trials (Schmidt-Pogoda et al., 2020). Funnel plot asymmetry and trim-and-fill analyses showed publication bias in preclinical and early phase trials, and the mean power of experimental studies was 17%. Less than half of preclinical studies reported randomization, <60% reported blinded outcome assessment, and sample sizes were smaller in a step-wise fashion between the three study types, all contributors to poor replicability.

Hylander and Repasky give a valuable set of recommendations in their careful discussion of how housing temperature can affect results in mouse models of cancer, immunity/inflammation, metabolism, adrenergic stress, energy homeostasis, and other physiological measures (Hylander and Repasky, 2016). They recommend that proper reporting practices be widely implemented for animal research, including the specification of environmental parameters such as housing, handling, food, lighting, and noise, as these affect behavior and brain chemistry. They state that while these parameters may be considered “seemingly mundane details,” they can potentially have large effects on experimental results. I agree that replicability in animal research would be helped by transparent reporting of environmental parameters, protocols, and raw data, rigorous sample size estimation, group randomization, blind outcome assessment, multiple testing correction, and certainly the ceasing of engagement in QRPs, issues I return to later when discussing solutions.

I assert that animal research-specific barriers to replicability (e.g., effects of housing temperature, site, strain) support the urgency of removing the incentives to engage in QRPs simply because the compounding of the former and the latter is far worse than the former alone. A critical addition to changing the incentives in animal research is the enforcement of comprehensive reporting guidelines that can enable scientists to most correctly replicate studies, and to understand potential influences on experimental results more generally. While I have focused on preclinical animal research, these recommendations would be beneficial to solving issues of replicability related to in vitro research as well (Hirsch and Schildknecht, 2019).

## Solutions

I contend that the two most practical steps to be taken that will have the greatest impact in improving the replicability, transparency, and overall quality of animal research are the widespread adoption of (1) registered reports, and (2) comprehensive reporting guidelines. Registered reports carry all of the benefits of preregistration for higher-quality science, with incentivized benefits for scientists, journals, and funding sources. The major distinction between preregistration and registered reports is that registered reports guarantee a publication, provided that the researchers adhere to preregistered experimental protocols that have been granted in-principle-acceptance. Additionally, because of the variability seen in the results of animal research across laboratories and animal strains, comprehensive reporting guidelines will enable researchers to control for as many extraneous factors as possible to maximize the chance of replicating and building on previous results.

## Registered Reports

Preregistration is a good scientific practice, but cannot alone realign scientific incentives, whereas registered reports can. Preregistration is the act of registering all experimental and analysis plans (hypotheses, design, protocols, analysis methods) before conducting a study. There have been several discussions about whether animal studies should be preregistered in the same manner as clinical trials to improve transparency, reduce misconduct, and improve replicability (Baker, 2019; van der Naald et al., 2020, 2022; Naald et al., 2021). I fully support the preregistration of animal studies, but preregistration as a scientific practice that improves transparency and replicability does not carry strong enough incentives to become adopted by the scientific community. In contrast, registered reports are an implementation of preregistration where a study plan (rather than a completed study) is submitted to a journal for peer review (Phase 1; Chambers and Tzavella, 2022). During Phase 1 peer review, reviewers evaluate the scientific question, experimental design, analysis methods, and all other methodological factors of a study. If a plan is agreed to between the scientists and the journal, it is preregistered with the journal and given the status of in-principle-acceptance, that is, the journal agrees to publish the completed study regardless of the results, as long as the study adheres to the preregistered plan. After the study is completed, the final manuscript is submitted to the journal only to confirm that it adhered to the preregistered study protocols (Phase 2). Phase 2 is designed to be shorter and more straightforward than Phase 1 and is followed by final acceptance and publication. The primary benefit of registered reports is the realignment of incentives away from positive findings that encourage engagement in QRPs, and toward answering relevant scientific questions with rigorous methodology.

The emphasis on questions and methods rather than results removes the incentives to p-hack, HARK, selectively report, and engage in QRPs more generally. Nonpreregistered experiments and analyses can still be included in a separate Results section, and the idea that registered reports limit creativity or exploration is a common misconception. When a study is completed and written up, the results of the preregistered analyses are included, with optional inclusion of nonpreregistered analyses. Nonpreregistered analyses conducted after new ideas arise from each piece of collected data are allowed. Registered reports do not aim to prohibit analytical or experimental flexibility, indeed much of biomedical research is exploratory, but rather to enable readers to evaluate the severity of all experimental and statistical tests, and part of this effort is to clearly define which tests were and were not preregistered. Deviations from preregistered analysis plans are not problematic in and of themselves; deviations that increase type 1 error rates (e.g., multiple testing without correction, stopping or continuing data collection based on statistical significance, selective outlier removal) and misrepresent the epistemic status of claims (e.g., HARKing) are. Frequently asked questions about registered reports are answered in a 2014 editorial and a recent comprehensive review (Chambers et al., 2014; Chambers and Tzavella, 2022)

Preregistration alone is an important tool to prevent excessive analytical flexibility after results are collected, but because of current incentives for positive findings, many researchers do not want to take the risk of being stuck with null findings that may be difficult to publish. Registered reports are the solution to this issue because researchers are assured a publication, enabling the primary focus to be placed on designing a study to rigorously answer an interesting scientific question, using protocols agreed on jointly between researchers and peer reviewers.

It is important to note that not all types of publications are suitable for registered reports. Studies that are purely exploratory with no hypotheses being tested do not need to be registered reports, nor do studies concerned with methods development. Registered reports are meant to be used for hypothesis-driven, confirmatory research.

Preregistration helps to keep experiments honest by disallowing excessive analytical flexibility, HARKing, and other QRPs. But again, preregistration collides directly with the incentives to obtain positive results. The major obstacle to preregistration becoming standard practice is the emphasis on positive findings in scientific publishing. I contend that preregistration is essential to improving the rigor and replicability of animal research, that the most pragmatic implementation of preregistration is registered reports, and that registered reports benefit researchers, journals, and funding sources. In the context of a registered report, researchers no longer have to worry about obtaining a positive result because the focus is placed entirely on answering a relevant scientific question with rigorous methodology. Hence, there is no incentive to engage in QRPs. For journals, peer review is more streamlined because Phase 1, when successful, declares a methodology to be used, and Phase 2 only confirms compliance with this methodology. For funding sources, registered reports would likely reduce the amount of funds wasted on false positive studies, increase the amount of overall disseminated science in the form of null findings (preventing future repeats of null studies), and positive data would likely be less contaminated by QRPs. Funding models may emerge where funding is dispensed simultaneously with in-principle-acceptance for a registered report (Chambers and Tzavella, 2022).

Along with removing incentives for researchers to engage in QRPs, registered reports protect researchers from peer reviewers who dislike their experimental design or ask for additional experiments, a burden especially for early career researchers with less disposable funding. Once a study is accepted in principle before data collection, the only requirement for publication is that researchers do not deviate from the preregistered study plan. Registered reports would reduce the amount of time a study is in peer review after data collection, and reduce the burden on researchers in their efforts to publish. Different publishing models for registered reports are emerging such as the Peer-Community In Registered Reports, a researcher-run platform that can peer review and help facilitate the completion of registered reports, with subsequent acceptance to a journal without further peer review (Eder and Frings, 2021).

Since preregistration and registered reports have seen growing adoption for roughly 20 and 10 years, respectively, recent work has compared the results of preregistered studies to studies of related topics that are not preregistered.

## The Growing Track Record of Registered Reports

There are patterns emerging on the impact of preregistration and registered reports on subjectively-related research quality, frequency of reported positive results, and replication success. As more registered reports are published, inferences can be made about the kinds of results they report, the quality of the studies themselves, and how they compare to nonpreregistered studies of similar topics. A recent study (Soderberg et al., 2021) recruited 353 researchers to peer review and compare pairs of psychology and neuroscience papers where one was a registered report (out of 29 total) and one was a standard report (57 total). Pairs were constructed to address similar topics, with half having the same first or corresponding authors or being published in the same journal. Papers were evaluated on 19 criteria measured before knowing study outcomes (8), after knowing study outcomes (7), and after finishing the paper (4). There were no differences in rated novelty and creativity, but large differences in rigor of methodology and analysis, along with overall paper quality in favor of registered reports. Nonpreregistered studies did not score higher than preregistered studies on any of the 19 criteria. This study gives preliminary evidence that registered reports are at least not of lesser quality than standard reports, and may be of higher quality in certain respects.

Results published in registered reports tend to show fewer positive findings than in standard reports. In a comparison of the frequency of positive results in psychology studies between registered reports and a random sample of standard reports, 44% of results in registered reports were positive, compared with 96% in standard reports (Scheel et al., 2021). In a comparison of effect sizes published in preregistered (93 effects) versus standard (900 effects) psychology studies, the mean and median effect size for preregistered effects were 0.21 and 0.16, and for standard reports, 0.4 and 0.36 (Schäfer and Schwarz, 2019). Effect sizes were mediated by study sample size in that larger sample sizes yielded smaller effects in both preregistered and standard reports, but less so in preregistered reports. This halving of effect sizes between standard and preregistered studies matches that seen in the large-scale replication projects of psychology studies.

Looking at the results of randomized controlled trials funded by the National Heart, Lung, and Blood Institute, 57% of studies published before 2000 reported significant improvements on primary outcomes, compared with 8% of studies published after 2000, when preregistration of primary outcomes became mandatory for clinical trials after Congress passed the Food and Drug Administration Modernization Act in 1997 (Kaplan and Irvin, 2015; ClinicalTrials.gov, 2021). This policy change showed the strongest association with the decline in positive findings, and neither increased use of active comparators (i.e., comparing interventions to one another rather than placebos) or decreased industry sponsorship showed this association. These results demonstrate that when researchers are incentivized to not deviate from a prespecified study plan, the frequency of positive results is often much lower.

Whether the replicability of registered reports (or preregistered studies in general) is higher than standard reports is an open question (Chambers and Tzavella, 2022), however evidence is emerging that this is the case. In a replication of 16 studies with “best practices” in place (i.e., preregistration, high statistical power to detect minimum effect sizes of interest, open methods), 86% of replications had statistically significant effects in the same direction as the original studies, and effect sizes were 97% of the originals (Protzko et al., 2020). Registered reports also show a similar percentage of null findings in both original research and replications. Allen and Mehler found that registered reports of original research were composed of 54.5% null findings, and registered reports of replications 66%, in contrast to the 5–20% of null findings in standard reports (Allen and Mehler, 2019).

## Applying Registered Reports to Sequences of Dependent Experiments in Neuroscience

A common misconception about registered reports is that they are only applicable in contexts where a single hypothesis is being tested (Chambers and Tzavella, 2022). An important consideration is how registered reports could be implemented for studies that include sequences of experiments that are dependent on one another. As an example, suppose a study is based on pilot data supporting a genetic perturbation that increases neuronal activity in a particular brain region of interest. To move forward, three potential experiments could test whether this increase of activity is resulting from differences in intrinsic excitability, increases in excitatory synaptic drive, or decreases in inhibition, or a combination of these. This represents at least seven distinct hypotheses, and each of these alternatives may logically preclude a different follow-up experiment. If a difference is seen in excitatory synaptic drive, the follow-up may be to determine whether this is presynaptic or postsynaptic (or both), yielding three hypotheses. If the change in drive is postsynaptic, it could be tested whether this is accompanied by differences in receptor number or ratios, synapse size, dendritic spine density, or other features. The combinatorial space for a sequence of experiments that each test multiple hypotheses can grow very quickly, and different experiments will likely have different requirements for adequate statistical power and positive/negative controls.

Three approaches to publishing an investigation of this kind as a registered report are (1) preregistering a decision tree of experiments with an if-else structure, with corresponding analysis plans for distinguishing between multiple alternative hypotheses; (2) iterative Stage 1 submission of individual, subsequent experiments after Stage 2 acceptance of previous experiments; and (3) including nonpreregistered studies as preliminary experiments, and only preregistering the final experiment, which would be using registered reports for a single experiment rather than multiple experiments.

In the context of preregistering a decision tree, different potential outcomes are associated with specific analysis plans, decision rules, effect sizes of interest, interpretations, statistical assumptions, and follow-up experiments. The preregistration may also include an outcome where the results do not align with any of the preregistered potential outcomes, necessitating a different approach to the subsequent experiment that would require a separate Stage 1 submission. Exploratory work can be added at any point in the decision tree.

**Figure 1.**
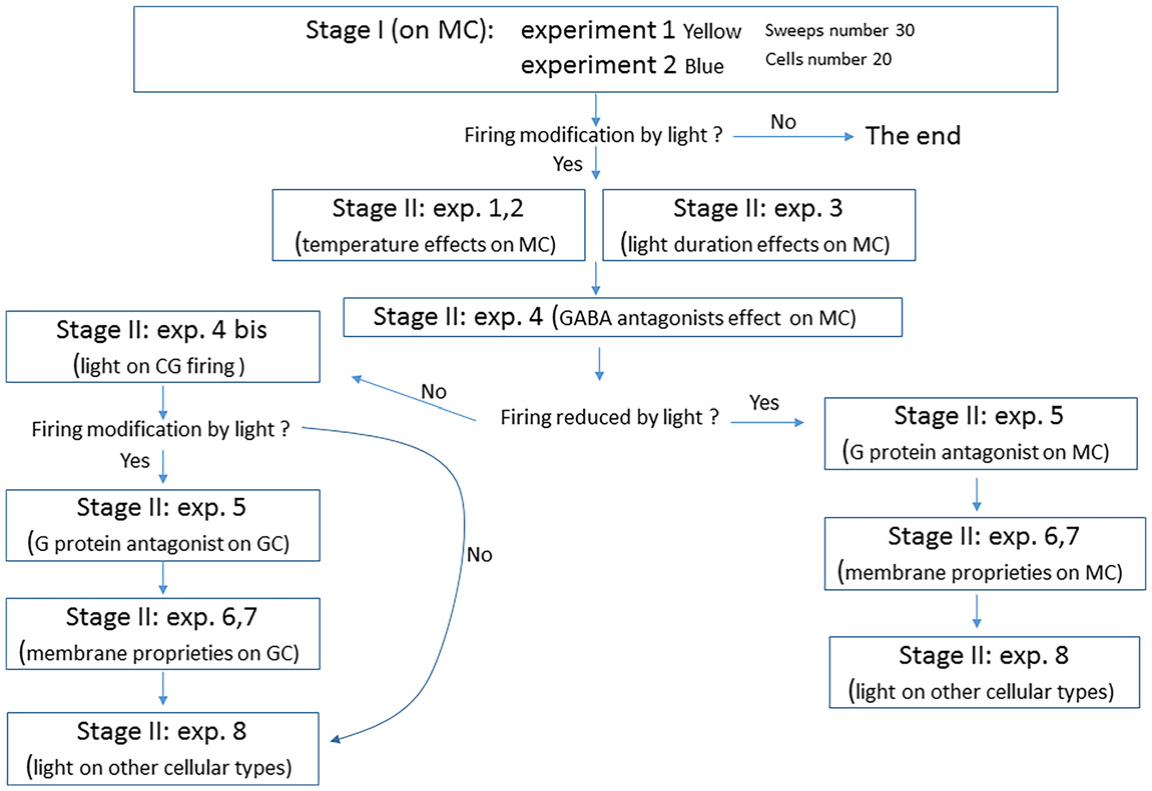
Flowchart of proposed experiments in the Stage 1 submission of Ait Ouares et al. (2019; reproduced with permission). “MC = mitral cells; CG = granular cells. The number of sweeps and cell recorded for the experiment performed on MC will be calculated depending on the outcome of Stage I experiments. The number of sweeps and cell recorded for the experiment performed on GC will be calculated depending on the outcome of Stage II experiments 4 bis.”

An example of this approach is a registered report by Ait Ouares et al. (2019; Fig. 1) of how light stimulation parameters typical of optogenetics experiments can affect neuronal firing in three cell types of the olfactory bulb and striatum in mice, when external opsins are not expressed (Ait Ouares et al., 2019). In their preregistered protocol (Ait Ouares et al., 2018, p. 1), pilot data are used to support two stages of proposed experiments. Stage 1 aimed at reproducing and expanding on their pilot data across two experiments, and Stage 2 included eight novel experiments that were dependent in several ways on those from Stage 1 that could easily generalize to many contexts within neuroscience. In these 8 experiments, two had two parts (E3 and E4), and the authors made clear that E4P2, and E6-E8 were exploratory and did not include power analyses for them. A flowchart in their protocol described the dependencies and decision rules between these experiments and choice of parameters. Briefly, the first three Stage 2 experiments explored the impact of light stimulation on slice temperature (E1), mitral cell firing (E2), and the dependence of changes in firing on stimulation duration (E3). Next, the decision tree becomes relevant. E4 examined the contribution of GABAergic inhibitory interneurons to these effects in mitral cells through the administration of GABAA and GABAB receptor antagonists. If E4 showed that GABA antagonists block the effects of light stimulation on mitral cell firing, then E4P2 would be a repeat of E4 but in granular cells. The subsequent experiments would examine the contribution of G-protein-coupled receptors (GPCRs) to these effects in granular cells (E5), the effects of light stimulation on properties of membrane potential and action potentials (E6–7), and the effects of light on other cell types (tufted cells in olfactory bulb, medium spiny neurons in striatum, cerebellar Purkinje neurons, and hippocampal CA1 pyramidal neurons; E8). If E4 showed that GABA antagonists do not block the effects of light stimulation on mitral cell firing, then E5-8 would focus on the effects of G-protein antagonists on light-induced mitral cell firing (E5), mitral cell membrane properties (E6-7), and the effects of light on firing in other cell types (E8). E4 did show that GABA antagonists did not block the effects of light on mitral cell firing, so subsequent experiments focused on mitral cells. All data and figures are clearly labeled as representing exploratory experiments and analyses. This study demonstrates that sequences of dependent experiments can be preregistered as a decision tree where experimental results determine the choice of subsequent experiments.

In the context of iterative Stage 1 submissions, the first Stage 1 submission and in-principle acceptance will likely take longer than those for each additional experiment. The initial submission establishes the background and focus of a study, and each additional experiment will only include the rationale, potential outcomes, and corresponding interpretations for one experiment, keeping the amount of work limited for researchers and reviewers. Additionally, while the preregistration for a subsequent experiment is being prepared or reviewed, pilot data may be collected to support the preregistration of this experiment, with the preregistration serving as a replication of the pilot data, similar to the two-stage process being piloted at the *Journal of Neuroscience* (Society for Neuroscience, 2020). For any iteration, exploratory tests that were not preregistered can be conducted to inform the preregistration of the subsequent experiment.

In the context of including nonpreregistered experiments as support for a subsequent preregistered experiment, we can use as an example a study by Heycke, Aust, and Stahl of different theories of preference acquisition (Heycke et al., 2017). Here, three experiments are presented, the first two being unregistered, the third being preregistered. Experiment 1 had a power analysis and sample size justification based on effect sizes from the literature, and experiment 2 tested the generalization of experiment 1. The purpose of experiment 3 (preregistered) was to replicate the findings of experiments 1 and 2 in a larger sample, with additional exploratory analyses about the sensitivity of results to different participant exclusion criteria and the order of dependent variables. The article includes a discussion of the limitations of all three experiments, and how the results of the unregistered experiments impacted specific design decisions in the preregistered experiment. This study demonstrates that registered reports are applicable to contexts where only the last experiment is preregistered, with prior studies included as unregistered support for the preregistered experiments. A study of this kind could be the first preregistration in a sequence of preregistrations as described in the second approach.

Regardless of the final arrangement for any particular study, at a minimum, hypotheses are specified to prevent HARKing, and results will not be subject to publication bias, which will likely increase the proportion of published null data as has been shown previously (Allen and Mehler, 2019; Scheel et al., 2021). On the other hand, registered reports and preregistration more generally are not meant to be straitjackets that prevent exploratory or serendipitous research. On the contrary, these approaches necessitate the explicit designation of hypotheses that are confirmatory or exploratory, clarifying the contribution of results from each.

If the trend of low statistical power (Button et al., 2013a; Dumas-Mallet et al., 2017) conflicts with the process of registered reports, where studies are not granted in-principle acceptance because they are underpowered, this would be a benefit as it would raise the standard for statistical power in neuroscience research. Registered reports certainly do not prohibit exploratory research, but confirmatory research must meet the standard of being adequately powered.

## Comprehensive Reporting Guidelines in Animal Studies

Along with the benefits of registered reports for improving replicability by removing incentives to engage in QRPs, I contend that animal research would benefit tremendously from more comprehensive reporting of genetic, environmental, and other variables that can influence study outcomes. Documenting environmental variables and sources of sensitivity is critical for improving the replicability and generalizability of animal research (Weissgerber et al., 2016). Sets of reporting guidelines have been proposed, including the ARRIVE 2.0 guidelines (Animal Research: Reporting of In Vivo Experiments; Percie du Sert et al., 2020), PREPARE (Planning Research and Experimental Procedures on Animals: Recommendations for Excellence; Smith et al., 2018), and others (Landis et al., 2012; Medical Research Council, 2015; Han et al., 2017; Bert et al., 2019; Smith, 2020; Gladman, 2021). The ARRIVE guidelines consist of a set of Essential 10 pieces of information that are proposed as a minimum requirement for reporting of animal studies, along with a Recommended Set of 11 other points that would ideally be integrated into publishing practice after the Essential 10 are established.

The Essential 10 consist of: study design [control/experimental groups and units (e.g., animal, litter, cage of animals)]; sample size (units per group, experiment total, sample size justification/calculation); inclusion/exclusion criteria (including number and justification of exclusions); randomization (method used and strategy for minimization of confounders); blinding; outcome measures; statistical methods (including software used, methods to assess whether statistical assumptions were met and alternative strategies if not); experimental animals (including health/immune status, genetic modifications, genotype, previous procedures); experimental procedures (including controls, enough detail to replicate, and the what/where/when/why of them); results (summary/descriptive statistics for each group, including a measure of variability, and an effect size with a confidence interval). The ARRIVE Essential 10 include all of the reporting guidelines advocated by the National Institute of Neurologic Disorders and Stroke after their 2012 meeting (Landis et al., 2012). The ARRIVE Recommended Set consists of: recommendations for information included in abstracts and background sections of manuscripts; clearly stated objectives; ethical statements; housing and husbandry conditions; animal care and monitoring; interpretation and scientific implications of findings; descriptions of generalizability and translation; protocol registration; data access statements; declarations of interest.

The PREPARE guidelines, meant to aid in planning studies (in contrast to the ARRIVE guidelines for reporting studies), consists of 15 items: literature searches; legal issues; ethnical issues, harm-benefit assessment, and human endpoints; experimental design and statistical analysis; objectives and timescale, funding, and division of labor; facility evaluation; education and training; health risks, waste disposal and decontamination; test substances and procedures; experimental animals; quarantine and health monitoring; housing and husbandry; experimental procedures; humane killing, release, reuse or rehoming; necropsy (Smith et al., 2018).

These two sets of guidelines share several points and provide a valuable roadmap to informing readers of animal studies of specific details that are important for conducting follow-up studies and replicating results. Their enforcement would be valuable to animal research, and while mostly incumbent on editorial and journal staff, all animal researchers should be aware of these guidelines as they evaluate new literature.

Many of the recommendations in the ARRIVE and PREPARE guidelines are endorsed in a recent report from the NIH Advisory Committee to the Director Working Group on Enhancing Rigor, Transparency, and Translatability in Animal Research (Gladman, 2021). The report focuses on five themes: (1) improving study design and data analysis; (2) addressing incomplete reporting and QRPs; (3) improving selection, design, and relevance of animal models; (4) improving methodological documentation and results reporting; (5) measuring and evaluating the costs and effectiveness of these four efforts. For improving study design and data analysis, the report recommends more statistical training and collaborations between animal researchers and statisticians, along with the creation of avenues in the NIH grant process to facilitate expert feedback on investigators’ study plans. For addressing incomplete reporting and QRPs, the report recommends increasing awareness about preregistering studies and the launching of pilot programs for preregistration and registered reports. For improving selection, design, and relevance of animal models, it is recommended that investigators justify their choice of animal model, exchange best practices for animal models, and that the NIH fund comparative animal-biology studies, along with public education of the benefits of animal research. For improving methodological documentation and results reporting, the report recommends increasing awareness among researchers of environmental factors that can affect research outcomes and should be documented, and supporting researchers in documenting long-term care of larger and long-lived animals. The report explicitly recommends the use of the ARRIVE Essential 10 checklist for reporting animal studies, along with statistical measures of uncertainty and effect size when reporting results.

I support the efforts of the NIH to bring attention and pilot solutions to these issues, and those of journals to enforce consistent reporting guidelines for animal studies that enable researchers to conduct precise replication and rigorous follow-up studies. Comprehensive reporting guidelines are a critical tool in facing the challenges arising from “seemingly mundane details” (Hylander and Repasky, 2016; e.g., housing temperature, lighting conditions, animal strain) that may influence the replicability and generalizability of scientific findings.

## Adversarial Collaborations

Another fascinating development that would resolve the problematic incentives for positive data are the use of adversarial collaborations (Mellers et al., 2001). Here, two or more opposing parties design and conduct a set of experiments to resolve some scientific debate, sometimes with the involvement of a neutral separate party. The opposition among involved parties provides a set of checks and balances to prevent analytical flexibility, publication bias, confirmation bias, and QRPs more generally which may coerce experimental results to suit a particular narrative. While interpretations of the final results may differ among the parties, the experimental design and data analysis are agreed on before any data are collected. In neuroscience, an adversarial collaboration is currently taking place to test two competing theories of consciousness: global neuronal workspace theory, and integrated information theory (Melloni et al., 2019, 2021). This adversarial collaboration is making use of multiple neuroimaging modalities (functional magnetic resonance imaging, electrocorticography, and magnetoencephalography simultaneous with electroencephalography) to study conscious vision in adult human participants. The study will consist of two preregistered experiments, designed by neuroscientists and philosophers, conducted across six independent laboratories, with open data and protocols, along with internal replications in separate subject samples. The leaders of this project frame it as building on the successes of “big science” approaches to answering big questions as exemplified by efforts in physics such as the Large Hadron Collider at the European Organization for Nuclear Research (CERN) or the Laser Interferometer Gravitational-Wave Observatory (LIGO), with counterparts in neuroscience such as the Allen Institute for Brain Science.

## Other Strategies

While registered reports would be effective for realigning academic incentives, comprehensive reporting guidelines for enabling scientists to replicate/generalize studies, and adversarial collaborations for pitting proponents of theories against one another to reduce bias, there are other scientific practices that may improve the quality of studies in animal research.

A further development that aims to improve the replicability and transparency of results reporting is machine-readable documents that store hypotheses, data, statistical results, decision rules, and other information in an easily reusable format that is useful for other scientists who want to examine the data, and meta-researchers who could more easily collate data about entire topics or fields (Lakens and DeBruine, 2021).

It is worth mentioning that along with QRPs and low statistical power contributing to low replicability, low prestudy odds strongly contribute as well (Wilson and Wixted, 2018; Ulrich and Miller, 2020). If a field predominantly tests incorrect hypotheses but publishes false positive findings, then replications are more likely to be unsuccessful. Additionally, studies with a large number of tested relationships without any selection procedures have lower positive predictive value (Ioannidis, 2005).

## Recommendations

I conclude with a short set of recommendations (Table 2) to improve replicability in neuroscience, all of which have been advocated previously (Simmons et al., 2011; Munafò et al., 2017; Chambers and Tzavella, 2022). The first and most important is that registered reports be widely adopted because of their removal of incentives to engage in QRPs, in turn improving the quality of published studies, and the safety net they provide researchers who may obtain null findings in assuring publication. Implicit in the recommendation of adoption of registered reports is prevention of analytical flexibility, prespecified stopping and data removal rules, adequate statistical power and sample size justification, reporting all dependent variables, experimental conditions, and animals/participants, and separation between confirmatory and exploratory analyses. Registered reports will also make peer review much easier on researchers, and this will be most valuable for early career researchers who may not have the resources to satisfy reviewer requests. Second, I advocate the adoption and enforcement of comprehensive reporting guidelines (e.g., ARRIVE, PREPARE) for animal studies which will enable researchers to most carefully and precisely replicate and generalize scientific findings. Third, I recommend greater openness and transparency in the form of sharing raw data, detailed protocols, analysis code, reagent information, and other materials. Fourth, greater importance should be placed on methodological and statistical training about the influence of p-hacking and other QRPs on false positive rates, what questions different statistics (e.g., *p*-value, effect size, Bayes factor) can and cannot answer, statistical power and sample size justification, multiple testing correction, blinded outcome assessment, randomization, and positive predictive value. Fifth, we must incentivize replication studies and adversarial collaborations to severely test old and new hypotheses and theories through public and private funding models. Finally, I recommend a greater awareness and valuing of practices that promote openness and replicability when hiring or promoting scientists, as they typically do not influence these decisions (Rice et al., 2020).

**Table 2:** Issues and recommendations to improve the replicability of neuroscience, and their recent developments

Problem	Solution	Developments/examples
QRPs (e.g., p-hacking, publication bias)	Registered reports improve incentives while preserving career advancement	>300 journals^1^; PCI-RR; ACD (theme 2)
Lack of detail to replicate/generalize studies	Adopt and enforce comprehensive reporting guidelines	ARRIVE; PREPARE; ACD (theme 4)
Lack of methods transparency	Share protocols, raw data, and analysis code	OSF; ACD (theme 5)
Incorrect analyses (e.g., identifying sex differences)	Improve methodological and statistical training and facilitate collaborations between experimental scientists and statisticians	ACD (theme 1)
Lack of incentives to replicate studies, test theories through adversarial collaborations	Incentivize replication studies and adversarial collaborations through funding opportunities	GAC
Lack of incentives to engage in open, pro-replication practices	Reward scientists who engage in scientific practices of openness, transparency, and rigor	Hiring, promotion^2^

ACD: Advisory Committee to the Director of NIH Working Group on Enhancing Rigor, Transparency, and Translatability in Animal Research (Gladman, 2021); PCI-RR: Peer Community In-Registered Reports (Eder and Frings, 2021); ARRIVE: Animal Research: Reporting of In Vivo Experiments (Percie du Sert et al., 2020); PREPARE: Planning Research and Experimental Procedures on Animals: Recommendations for Excellence (Smith et al., 2018); OSF: Open Science Framework; GAC: Generative Adversarial Collaborations (2021; Retrieved April 24, 2022, from https://gac.ccneuro.org/); CoS: Center for Open Science.

1 https://www.cos.io/initiatives/registered-reports.

2 (Rice et al., 2020).

## Conclusion

Evidence from large replication projects clearly demonstrates that replicability is low across scientific fields. Factors contributing to low replicability include QRPs, misunderstanding of *p*-values, and low statistical power. One of the most significant factors in low replicability is academic incentives to engage in QRPs that increase the rate of published false positive findings. All of these issues are present in animal research, the most striking of which are the low publication rate of animal studies, low rates of blinded outcome assessment and randomization, low statistical power, and high rate of statistically significant findings. While the awareness of QRPs goes back centuries, only in the past 10–15 years have we obtained empirical data from large-scale replication projects demonstrating that replicability is far from ideal. This confers a responsibility on the current generation of scientists to do something about it. Two of the most practical and impactful steps to be taken in combating low replicability and improving overall research quality in animal research are the widespread adoption of registered reports and comprehensive reporting guidelines. Registered reports realign academic incentives to answer scientifically relevant questions with rigorous methodology instead of obtaining positive results, and comprehensive reporting guidelines inform researchers about study parameters that can have significant effects on experimental results.

One of the goals of this commentary is to galvanize conversations about QRPs, registered reports, statistical power, and replicability more generally among animal researchers. To date, the vast majority of discussion of these issues has been among psychologists and statisticians, and I hope these trends spread to other fields. I applaud the growing awareness of these issues among animal researchers as can be seen in a recent editorial in the *Journal of Neuroscience* (Society for Neuroscience, 2020), a May 2021 special issue of *Animal Behavior and Cognition* about replicability in animal behavior research (Brecht et al., 2021), and innovative ideas about using historical control group data to increase statistical power (Bonapersona et al., 2021). I also applaud the growing movement toward registered reports, with over 300 journals offering them at the time of writing (Center for Open Science, 2022), and recent calls for their widespread adoption on ethical grounds that they would help science more effectively serve society (Van Calster et al., 2021).

Some may argue that the rate of ongoing scientific progress is adequate, and that scientific practice does not require systemic modification to improve replicability. Recent scientific achievements like the swiftly developed COVID-19 vaccines, CRISPR, and declining cancer mortality rates are all impactful, but this picture excludes the counterfactual that if replicability were improved, the time required to make these advancements and the suffering and waste accrued along the way would be reduced. How many lines of research have been pursued based on prior p-hacked, HARKed, or otherwise low-quality studies and ended in failure, wasted time and resources? How many lives have been and are going to be lost to the pursuit of doomed-to-fail research programs that are based on false positive findings tainted by QRPs and low power? Arguments about long-run advancement and self-correction do not justify the waste of time, money, and loss of lives that could be saved if the scientific enterprise were to remove incentives to engage in QRPs, and was focused on questions and methods rather than results. There are clear, actionable, pragmatic paths forward to reduce the time to major scientific advancements, and I urge the scientific community to pursue them. It is incumbent on funding sources, government research institutions, universities, and individual researchers to improve replicability and the incentives of science to ensure proper use of and returns on public and private investment.

## Synthesis

Reviewing Editor: Mark Laubach, American University Decisions are customarily a result of the Reviewing Editor and the peer reviewers coming together and discussing their recommendations until a consensus is reached. When revisions are invited, a fact-based synthesis statement explaining their decision and outlining what is needed to prepare a revision will be listed below. The following reviewer(s) agreed to reveal their identity: Arvind Kumar, Guillaume Rousselet.

Two expert reviewers reviewed your study. Their reviews are given below. Please address all points that were raised. Thank you for submitting your study to eNeuro!

Some comments from our discussion of your paper are relevant to how you proceed. Please review the comments below carefully and in light of the reviews posted below.

There was agreement that the manuscript is a good summary, without novelty, of registered report but was not specific enough to sub-fields of neuroscience. Given the amount of work that went into the manuscript, it was felt that there is potential for a much better revised version: shorter, restructured by theme, and with case studies and recommendations specific to preclinical work.

The manuscript hinges on the two aspects i.e. registered reports and complete reporting. But these are already in practice. You must go beyond the current state of this topic and provide new suggestions/ideas to improve how registered reports are being used.

The manuscript must be shortened and focused more on neuroscience.

Some suggestions are to include case studies or examples specific to preclinical neuroscience research. If examples specific to preclinical work cannot be identified, then you could conduct a survey of the literature in a few key journals, compare practices across sub-field in neuroscience, and conclude with brief recommendations and citations to relevant published reviews.

Reviewer 1

In this manuscript the author has provided an extensive review of questionable research practices (ORPs) and the problem of irreproducibility of scientific results. The author also discusses potential means to mitigate the problem of QRPs and reproducibility of scientific results.

The manuscript is rather long and much of the issues about QRPs and reproducibility of scientific results has been written about earlier (and cited well in this manuscript). A lot of discussion about social science, cancer biology, economics and psychology (especially in the first 15 pages or so) is not really relevant to the Neuroscience audience.

As a potential suggestion to the problem of QRPs and reproducibility of scientific results the authors suggest ‘widespread adoption of Registered Reports and Comprehensive reporting guidelines’. This is nothing new. These methods are already in place and their use is only increasing.

I do agree with the authors that we must speed up the progress towards quenching the QRPs and improve trust in scientific results but I am afraid the current manuscript only repeats what is already known or is being practiced. I am not sure how this will help in galvanizing the conversation about QRPs.

Some specific comments:

- The low percentage of publication of pre-registered studies does not automatically mean publication bias or selective reporting. There can be other reasons for lack of publications e.g. study may be left incomplete due to funding or staff issue or manuscript was not revised after reviewers’ comments. Is there any data on that? Van de Naald et al only speculate about selective reporting, as Theo wrote: “Although not as extensively evaluated, there is also reason for concern regarding the selective publication of preclinical animal studies.”

So I think the author should provide stronger arguments.

- The example of the Ouares et al for registered reports to sequence of dependent experiments is very nice. I think it may be useful to show the flowchart here in the manuscript.

- I think the important issue is how to define which research is hypothesis driven and which is exploratory. All exploratory research in principle is HARKing.

- Some important omissions that I think should be a part of the discussion:

1. Adversarial Collaboration to test GNW and IIT. https://osf.io/mbcfy/

I think this is a great strategy to avoid QRPs and ensure reproducibility and should be commented on.

2. Elber-Dorozko and Loewenstein elife 2018: Here authors discuss how the study of neural correlates of behaviour may run into not only erroneous but also irreproducible results.

Reviewer 2

Your article is well written and present a very good summary of issues and solutions. My main point is whether the content and structure are appropriate for the target audience. The text start with long descriptions of the issues, including detailed summaries of replication projects in psychology and other fields. We get to preclinical neuroscience only on page 17, but even in that section, most of the content is about other fields. If the goal is to educate animal researchers, a different structure could be more effective: in subsections, present issues and their solutions, first drawing from the broad psych/neuro literature, followed by specific case studies and recommendations for preclinical neuroscience. The text could be shortened in many places, as some of the reporting seems too detailed. Figures could help both lighten the text and educate. Without a better structure, more focused on preclinical research, I’m not sure your article will have the impact it deserves. As it stands, a few paragraphs and sentences about preclinical neuro could be deleted, and we would be left with a solid summary about QRPs, research culture, statistics and various solutions. It almost feels misleading to have preclinical animal research in the title, as there isn’t much specifically about the topic in the article. For inspiration, I recommend this great summary about issues leading to false positives, and remedies:

https://pubmed.ncbi.nlm.nih.gov/27879038/

The article uses figures and tables, and includes case studies from the fields of ecology and genetics.

Other points:

Clear definitions are needed, starting in the abstract. For instance, the terms replicability and reproducibility seemed to be used inter-changeably. I tend to think about the issue you describe as replicability. There is also a reproducibility issue, but that’s because researchers don’t share their data and code:

https://www.frontiersin.org/articles/10.3389/fninf.2017.00076/full#B14

https://www.biorxiv.org/content/10.1101/066803v1.full.pdf

About better reporting, this editorial is worth mentioning:

https://www.ncbi.nlm.nih.gov/pmc/articles/PMC5330667/

Generalizability - a key reference is missing; it presents solutions involving more rigorous data modelling, going beyond the standard yet generally inappropriate t-tests or rank-based equivalent used in animal research:

https://psyarxiv.com/jqw35/

Descriptions of power could be improved:

"these replications had high statistical power"

"Low statistical power introduces two important vulnerabilities to a scientific study"

"Neuroscience studies tend to be underpowered"

It should be clear that statistical power is a property of an infinitely long programme of research, not a single experiment.

"Mean statistical power”: “mean” should be deleted because power cannot be defined for a single replication.

"As a significant portion of science is publicly funded, it is incumbent upon funding sources, government research institutions, universities, and individual researchers to improve reproducibility to ensure proper use of and returns on public investment.”

and later on:

"Being that most science is publicly-funded, it is arguably unethical to use taxpayer money to publish QRP-ridden research.”

Irrespective of the source of funding, it is unethical to waste people’s time and animals’ life in hopeless research.

"most journals do not encourage the submission of replications” - and even when they do, they don’t necessarily act according to their editorial policies:

https://retractionwatch.com/2018/05/08/nature-says-it-wants-to-publish-replication-attempts-so-what-happened-when-a-group-of-authors-submitted-one-to-nature-neuroscience/

"A p-value represents the probability of obtaining a result, or one more extreme, on the condition of the null hypothesis being true.” - here “null” should be removed, as it is not part of the definition; a “point hypothesis” would be more accurate. Same issue in “statistical power is the probability that the null hypothesis will be rejected”.

A key reference about the interpretation of p values is missing:

https://link.springer.com/article/10.1007/s10654-016-0149-3

"Vasishth et al. called Type M and Type S”: the original reference is Gelman & Carlin. See also:

Shaffer, J. P. (2002). Multiplicity, directional (Type III) errors, and the Null Hypothesis. Psychological Methods, 7(3), 356-369.

"the effect size of statistically significant effects in low-powered settings are likely exaggerated or may not exist at all” - “or may not exist at all” should be deleted because an effect size necessarily exists.

"it is arguably most important for a field to know true effect sizes for effects that exist” — I don’t think “true effect sizes” is clear, here and in the next sentence. Could be replaced with “for a field to precisely estimate effect sizes”. I’m not sure we can ever know what the true effect size is. As you point out later, there are many co-variates (experimental variables such as lab temperature) that can affect effect sizes, so a true population effect size is an abstraction; it only exists in simulation work.

"this tendency yielded a statistically significant negative correlation” - this reporting seems to be at odd with your descriptions. Why not report the correlation + some measure of uncertainty without an arbitrary dichotomisation of the effect?

Similarly, results from Crabbe et al. 1999 are described using p values and mention of “statistically significant effects”. This is at odd with previous sections highlighting the dangers of filtering results by significance. Why even report p values in a research summary?

"Encouragingly, there were no differences across sites” - if this statement is based on p>0.05, it is a classic inferential fallacy. Differences across sites cannot be exactly zero.

"opposite significant,” — not sure what that means. Instead of the quotation marks of mystery, be specific instead.

Writing is good, except for the transitions between paragraphs, which sometimes lack clear and explicit logical links. Each paragraph should start with a clear description of its main message. For instance, the paragraph starting with the sentence “I briefly review some of the sensitivities observed in preclinical animal research.”, is followed by the descriptions of several studies across several paragraphs, but the main message is unclear, which makes the information difficult to digest.

"unknown sources of sensitivity” — sensitivity to what?

The papers by Heroux et al. and Luck & Gaspelin seem to be mostly about research in humans, but they are presented in a paragraph about animal research. This should be clear to the reader or the information should be restructured.

Signed review: Guillaume Rousselet

## Author Response

Author’s Rebuttal

I earnestly thank the Reviewers for their thorough and constructive criticisms. In this revision, I have addressed all criticisms and included new discussions that strengthen my Societal Impact piece about how insights from metascience can inform neuroscience, and why registered reports and comprehensive reporting guidelines are essential additions to the current neuroscience toolkit.

The single major criticism from the Editor and Reviewers was that not enough focus is placed on neuroscience in the first ∼15 pages, with most of the text up until then focused on other fields (e.g., psychology, cancer biology). To remedy this, I have added neuroscience-specific case studies and discussions to all sections composing the first ∼15 pages and removed excess content that is not neuroscience-focused.

Specifically, the first ∼15 pages include the introduction, along with discussions of large-scale replication projects, questionable research practices (QRPs) and academic incentives, misunderstanding of p-values, and low statistical power. I have added substantial neuroscience-specific discussions to the sections on large-scale replication projects and misunderstanding of p-values, with briefer additions to the section on QRPs because there is almost no neuroscience-specific literature in this area. The section on statistical power already included considerable neuroscience-specific discussions but has been revised according to the specific comments of the reviewers.

While the majority of metascience research has not focused on neuroscience, one of my goals is for this article to be a self-contained, comprehensive resource to introduce neuroscientists to metascience, and this goal is now made explicit in the Introduction. I contend that the non-neuroscience content is essential to accomplishing this goal, but I have taken care to remove unnecessary content from the manuscript.

Aside from this, I addressed all additional conceptual, explanatory, and technical concerns raised by the Reviewers, and believe the revised article is clearer and will be of much greater value to the readership of eNeuro and the wider scientific community.

Below is my point-by-point response to the Reviewers’ comments (in blue), with selections of longer comments bolded for clarity.

Reviewers’ comments:

Reviewer 1

In this manuscript the author has provided an extensive review of questionable research practices (ORPs) and the problem of irreproducibility of scientific results. The author also discusses potential means to mitigate the problem of QRPs and reproducibility of scientific results.

The manuscript is rather long and much of the issues about QRPs and reproducibility of scientific results has been written about earlier (and cited well in this manuscript). A lot of discussion about social science, cancer biology, economics and psychology (especially in the first 15 pages or so) is not really relevant to the Neuroscience audience.

As a potential suggestion to the problem of QRPs and reproducibility of scientific results the authors suggest ‘widespread adoption of Registered Reports and Comprehensive reporting guidelines’. This is nothing new. These methods are already in place and their use is only increasing.

I do agree with the authors that we must speed up the progress towards quenching the QRPs and improve trust in scientific results but I am afraid the current manuscript only repeats what is already known or is being practiced. I am not sure how this will help in galvanizing the conversation about QRPs.

Thank you for these valuable points. To maintain the article’s focus on neuroscience, I have added multiple neuroscience-specific discussions to the first 15 pages, highlighting problems and solutions in specific case studies with references. Because there have been no completed large-scale replication studies focused on neuroscience, I was limited to discussing findings from replication studies in other fields. In this revision, I have added discussions of published small-scale replication studies in neuroscience, in-progress large-scale replication and many-analyst projects in neuroscience, and adversarial collaboration projects in neuroscience. I have also added multiple case studies related to misuse of statistical testing when measuring sex differences, statistical interaction effects, and analyzing data from specific experimental techniques.

Some specific comments:

- The low percentage of publication of pre-registered studies does not automatically mean publication bias or selective reporting. There can be other reasons for lack of publications e.g. study may be left incomplete due to funding or staff issue or manuscript was not revised after reviewers’ comments. Is there any data on that? Van de Naald et al only speculate about selective reporting, as Theo wrote: “Although not as extensively evaluated, there is also reason for concern regarding the selective publication of preclinical animal studies.”

So I think the author should provide stronger arguments.

Thank you for this comment. I agree that low publication rates do not necessitate publication bias, and have added this qualification to the discussion of the van der Naald et al. and other studies to make this clear. I have also included additional arguments and references to support the notion that publication bias is a likely contributor to low publication rates in animal research (pp. 20-21).

Measuring the specific contribution of publication bias to low animal publication rates is difficult because it requires knowing the exact reason for publication or non-publication of specific animals. Two methods to estimate this contribution are meta-analyses assessing publication bias in animal research, and surveys of animal researchers. In 21 meta-analyses assessing publication bias, evidence was found in 13 (62%) based on Egger’s test for funnel plot asymmetry and trim-and-fill analysis. Additionally, across 16 systematic reviews of 525 animal studies describing interventions to treat acute ischemic stroke, only 1.2% found no statistically significant findings, and the same two methods (i.e., Egger’s test and trim-and-fill) indicated evidence for publication bias in 16 and 10, respectively, potentially accounting for 1/3 of reported efficacy.

Finally, a survey of 454 animal researchers estimated that 50% of animal experiments are published, in line with van der Naald et al. and other estimates of animal publication rates, but it also rated “Lack of statistically significant differences (’negative’ findings)” as the most important reason for non-publication. This finding aligns with another survey of psychologists (John et al., 2012; mentioned earlier in the manuscript) who admitted to “Selectively reporting studies that ’worked’” 45.8-50% of the time, while estimating the prevalence to be 60%.

In sum, while the evidence shows that publication rates of experimental animals are somewhere in the range of 50%, measuring the specific contribution of publication bias is less certain, though there is statistical and survey evidence indicating it as a potent contributor.

- The example of the Ouares et al for registered reports to sequence of dependent experiments is very nice. I think it may be useful to show the flowchart here in the manuscript.

Thank you for this comment. I reached out to the authors and they gave me permission via email to include their flowchart in my manuscript, and it is now included.

- I think the important issue is how to define which research is hypothesis driven and which is exploratory. All exploratory research in principle is HARKing.

Agreed this point is worth emphasizing. At the end of the first paragraph of the section, “Questionable research practices and perverse incentives,” I added the sentence: “It is worth noting that HARKing is only problematic in the context of confirmatory research, not exploratory research where hypotheses are not being tested, but rather generated.”

- Some important omissions that I think should be a part of the discussion:

1. Adversarial Collaboration to test GNW and IIT. https://osf.io/mbcfy/

I think this is a great strategy to avoid QRPs and ensure reproducibility and should be commented on.

Thank you for this comment.

Agreed this is an excellent innovation to safeguard against QRPs. I have added discussion of adversarial collaborations and this particular adversarial collaboration in the section, “Other Strategies.”

2. Elber-Dorozko and Loewenstein elife 2018: Here authors discuss how the study of neural correlates of behaviour may run into not only erroneous but also irreproducible results.

I have added discussion of this reference as a neuroscience case study in the section “Misunderstanding of p-values,” along with other neuroscience-specific case studies.

Reviewer 2

Your article is well written and present a very good summary of issues and solutions. My main point is whether the content and structure are appropriate for the target audience. The text start with long descriptions of the issues, including detailed summaries of replication projects in psychology and other fields. We get to preclinical neuroscience only on page 17, but even in that section, most of the content is about other fields. If the goal is to educate animal researchers, a different structure could be more effective: in subsections, present issues and their solutions, first drawing from the broad psych/neuro literature, followed by specific case studies and recommendations for preclinical neuroscience. The text could be shortened in many places, as some of the reporting seems too detailed. Figures could help both lighten the text and educate. Without a better structure, more focused on preclinical research, I’m not sure your article will have the impact it deserves. As it stands, a few paragraphs and sentences about preclinical neuro could be deleted, and we would be left with a solid summary about QRPs, research culture, statistics and various solutions. It almost feels misleading to have preclinical animal research in the title, as there isn’t much specifically about the topic in the article. For inspiration, I recommend this great summary about issues leading to false positives, and remedies:

https://pubmed.ncbi.nlm.nih.gov/27879038/

The article uses figures and tables, and includes case studies from the fields of ecology and genetics.

Thank you for these valuable points. Centering the article’s focus on neuroscience, I have added multiple neuroscience-specific discussions to the first 17 pages, highlighting problems and solutions in specific case studies with references. In addition, to concretely illustrate how registered reports can be used by neuroscientists, I have added one figure (reproduced with permission from the original authors) showing the decision tree of proposed experiments from a published neuroscience registered report. I have also added a table in the Recommendations section near the end of the article summarizing the problems, solutions, and recent developments discussed in the article as a convenient reference for readers. I have also removed a substantial amount of non-neuroscience content.

Other points:

Clear definitions are needed, starting in the abstract. For instance, the terms replicability and reproducibility seemed to be used inter-changeably. I tend to think about the issue you describe as replicability. There is also a reproducibility issue, but that’s because researchers don’t share their data and code:

https://www.frontiersin.org/articles/10.3389/fninf.2017.00076/full#B14

https://www.biorxiv.org/content/10.1101/066803v1.full.pdf

Thank you for this comment, and indeed in almost all uses of “reproducibility,” I meant “replicability.” All inappropriate uses of “reproducibility” have been replaced with “replicability,” and I give definitions of “reproducibility,” “replicability,” and “robustness” according to a recent review (Nosek et al., 2021) at the end of the Introduction.

About better reporting, this editorial is worth mentioning:

https://www.ncbi.nlm.nih.gov/pmc/articles/PMC5330667/

Thank you for this reference, it has been added to the first paragraph of the section, “Comprehensive reporting guidelines in animal studies.”

Generalizability - a key reference is missing; it presents solutions involving more rigorous data modelling, going beyond the standard yet generally inappropriate t-tests or rank-based equivalent used in animal research:

https://psyarxiv.com/jqw35/

Added this excellent reference to the discussion of generalizability.

Descriptions of power could be improved:

"these replications had high statistical power"

"Low statistical power introduces two important vulnerabilities to a scientific study"

"Neuroscience studies tend to be underpowered"

It should be clear that statistical power is a property of an infinitely long programme of research, not a single experiment.

"Mean statistical power”: “mean” should be deleted because power cannot be defined for a single replication.

I apologize, but I’m not sure I understand this comment. Could you please provide references about statistical power being a property of research programs rather than individual experiments? I tried searching but could not find any. I have only understood statistical power as a property to be calculated and justified before running experiments, in addition to justifying effect sizes of interest and sample sizes. Post-hoc power is the topic of the neuroscience-specific Button et al. review discussed in the section about statistical power, as well as the more general Dumas-Mallet review.

"As a significant portion of science is publicly funded, it is incumbent upon funding sources, government research institutions, universities, and individual researchers to improve reproducibility to ensure proper use of and returns on public investment.”

and later on:

"Being that most science is publicly-funded, it is arguably unethical to use taxpayer money to publish QRP-ridden research.”

Irrespective of the source of funding, it is unethical to waste people’s time and animals’ life in hopeless research.

Thank you, I fully agree and have modified these sentences to reflect these points.

"most journals do not encourage the submission of replications” - and even when they do, they don’t necessarily act according to their editorial policies:

https://retractionwatch.com/2018/05/08/nature-says-it-wants-to-publish-replication-attempts-so-what-happened-when-a-group-of-authors-submitted-one-to-nature-neuroscience/

Added this pertinent reference to the section, “Questionable research practices and perverse incentives.”

"A p-value represents the probability of obtaining a result, or one more extreme, on the condition of the null hypothesis being true.” - here “null” should be removed, as it is not part of the definition; a “point hypothesis” would be more accurate. Same issue in “statistical power is the probability that the null hypothesis will be rejected”.

Thank you, I replaced “null” with “point” in these sentences.

A key reference about the interpretation of p values is missing:

https://link.springer.com/article/10.1007/s10654-016-0149-3

Thank you for this reference, I added it to the first sentence in the section, “Misunderstanding of p-values.”

"Vasishth et al. called Type M and Type S”: the original reference is Gelman & Carlin. See also:

Shaffer, J. P. (2002). Multiplicity, directional (Type III) errors, and the Null Hypothesis. Psychological Methods, 7(3), 356-369.

Thank you for pointing this out. I replaced the Vasishth et al. reference in this sentence with Gelman & Carlin, and refer to Vasishth et al. in the following sentence.

"the effect size of statistically significant effects in low-powered settings are likely exaggerated or may not exist at all” - “or may not exist at all” should be deleted because an effect size necessarily exists.

Agreed; this clause has been removed from the sentence.

"it is arguably most important for a field to know true effect sizes for effects that exist” — I don’t think “true effect sizes” is clear, here and in the next sentence. Could be replaced with “for a field to precisely estimate effect sizes”. I’m not sure we can ever know what the true effect size is. As you point out later, there are many co-variates (experimental variables such as lab temperature) that can affect effect sizes, so a true population effect size is an abstraction; it only exists in simulation work.

Thank you for this point, this sentence now reads: “However, this point overlooks that the effect size of statistically significant effects in low-powered settings are likely exaggerated, and it is arguably most important for a field to precisely estimate effect size magnitudes, rather than only if they are statistically significant or not.”

"this tendency yielded a statistically significant negative correlation” - this reporting seems to be at odd with your descriptions. Why not report the correlation + some measure of uncertainty without an arbitrary dichotomisation of the effect?

Added more information about the three effect size categories examined (i.e., small, medium, large), along with the correlations and confidence intervals for all three categories.

Similarly, results from Crabbe et al. 1999 are described using p values and mention of “statistically significant effects”. This is at odd with previous sections highlighting the dangers of filtering results by significance. Why even report p values in a research summary?

These p-values are reported in addition to effect sizes (in this case, partial omega squared) to give full statistical descriptions of the results. I agree that filtering results only by significance is dangerous, and that it is essential to report effect sizes to evaluate whether effect sizes are meaningful rather than just statistically significant.

"Encouragingly, there were no differences across sites” - if this statement is based on p>0.05, it is a classic inferential fallacy. Differences across sites cannot be exactly zero.

Thank you for this comment. This sentence now reads, “Encouragingly, there were no statistically significant differences across sites for water maze escape latency or alcohol consumed.”

"opposite significant,” — not sure what that means. Instead of the quotation marks of mystery, be specific instead.

This language has been revised to “statistically significant in opposite directions.”

Writing is good, except for the transitions between paragraphs, which sometimes lack clear and explicit logical links. Each paragraph should start with a clear description of its main message. For instance, the paragraph starting with the sentence “I briefly review some of the sensitivities observed in preclinical animal research.”, is followed by the descriptions of several studies across several paragraphs, but the main message is unclear, which makes the information difficult to digest.

Thank you for this comment. I reviewed the first sentence of each paragraph and made appropriate modifications for clarity where needed.

"unknown sources of sensitivity” — sensitivity to what?

Thank you for this comment. This sentence has been revised to, “The high controllability of animal experiments is both a blessing and a curse—for the ability to control extraneous factors and identify biological mechanisms beyond what can ethically be done in humans, with the caveat that many factors (co-housing, food/water, strain) may still compromise the replicability and generalizability of results, respectively.

The papers by Heroux et al. and Luck & Gaspelin seem to be mostly about research in humans, but they are presented in a paragraph about animal research. This should be clear to the reader or the information should be restructured.

These references have been specifically described as referring to human studies.
